# Asymmetric synthesis of a highly functionalized bicyclo[3.2.2]nonene derivative

**DOI:** 10.3762/bjoc.9.74

**Published:** 2013-04-04

**Authors:** Toshiki Tabuchi, Daisuke Urabe, Masayuki Inoue

**Affiliations:** 1 Graduate School of Pharmaceutical Sciences, The University of Tokyo, Hongo, Bunkyo-ku, Tokyo 133-0033 Japan

**Keywords:** asymmetric synthesis, *C*_2_-symmetry, catalysis, Diels–Alder reaction, Lewis acid, natural product, quaternary carbon

## Abstract

The stereoselective Diels–Alder reaction between an optically active 1,4-dimethylcycloheptadiene and acrolein was effectively promoted by TBSOTf to produce a bicyclo[3.2.2]nonene derivative bearing two quaternary carbons. Seven additional transformations from the obtained bicycle delivered the *C*_2_-symmetric bicyclo[3.3.2]decene derivative, a key intermediate in our synthetic study of ryanodine.

## Introduction

**Scheme 1:**
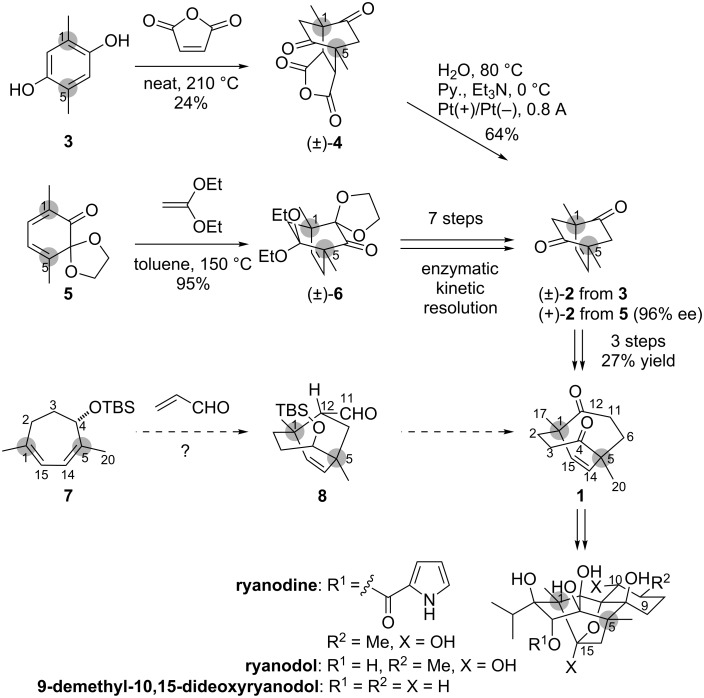
Structure of ryanodine and the Diels–Alder reactions for construction of the potential intermediates of ryanodine.

Ryanodine (Scheme 1) [1–3] is a potent modulator of the intracellular calcium release channels, known as ryanodine receptors [4–5]. Its complex architecture, including eight contiguous tetrasubstituted carbons on the pentacyclic ABCDE-ring system, has posed a formidable synthetic challenge. To date, the only total synthesis of a compound in this class of natural products was reported by Deslongchamps, who constructed ryanodol in 1979 [6–9]. Most recently, we reported the synthesis of 9-demethyl-10,15-dideoxyryanodol [10] by taking advantage of the intrinsic *C*_2_-symmetry of the target molecule. In this synthesis, *C*_2_-symmetric compounds such as bicyclo[3.3.2]decene **1** were strategically designed, and application of pairwise functionalizations of these molecules minimized the total number of steps.

Bicyclo[3.3.2]decene **1** was prepared from *C*_2_-symmetric bicyclo[2.2.2]octene **2** through a ring-expansion reaction (Scheme 1) [11]. We reported the synthetic routes to racemic **2** and enantiomerically pure **2** from **3** and **5**, respectively. Specifically, the dearomatizing Diels–Alder reaction between 2,5-dimethylbenzene-1,4-diol (**3**) and maleic anhydride lead to the construction of bicyclo[2.2.2]octene **4**, which was then transformed into racemic **2** through electrolysis [11]. Alternatively, the Diels–Alder reaction between 3,6-dimethyl-*o*-quinone monoacetal **5** and 1,1-diethoxyethylene provided bicyclo[2.2.2]octene **6**, which was then converted to enantiopure **2** via an enzymatic kinetic resolution [12]. The Diels–Alder reaction was effectively employed in both of these syntheses for construction of the two quaternary carbons at the C1 and C5 positions of ryanodine (highlighted in gray). However, the current route to (+)-**2** from racemic **6** generated the unnecessary antipode. Therefore, development of an alternative asymmetric route to **1** was planned to further improve the overall practicality. Here we report an asymmetric Diels–Alder reaction for simultaneous installation of the C1- and C5-stereocenters using the optically active cycloheptadiene derivative **7**, and its derivatization into bicyclo[3.3.2]decene **1**.

We assumed that the C4-stereocenter of optically active seven-membered diene **7** would permit the requisite stereoselective Diels–Alder reaction (Scheme 1). Namely, the reaction between **7** and acrolein was expected to stereoselectively introduce the C1, C5 and C12 stereocenters to afford bicyclo[3.2.2]nonene **8**. The C11-aldehyde of **8** was then to be utilized as a handle for the ring expansion to access **1**. To the best of our knowledge, construction of the two quaternary carbons by the intermolecular Diels–Alder reaction of 1,4-disubstituted cycloheptadiene derivatives has not been reported [13–14].

## Results and Discussion

**Scheme 2:**
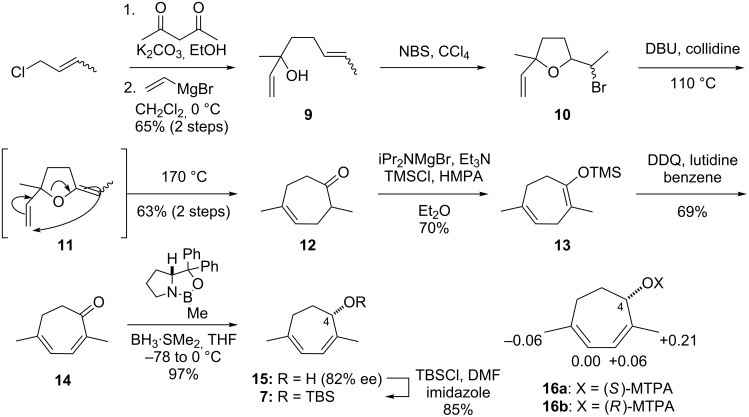
Asymmetric synthesis of **7** and determination of the absolute configuration at C4 of **15** by the modified Mosher method. The numbers are differences in ^1^H chemical shifts between **16a** and **16b** (Δδ = δ**16a** − δ**16b**).

The synthesis of optically active **7** began from crotyl chloride (Scheme 2). The carbon chain extension of crotyl chloride by treatment with acetylacetone and K_2_CO_3_ [15], followed by the addition of vinylmagnesium bromide [16], provided **9**. The bromoetherification of tertiary alcohol **9** by using NBS led to tetrahydrofuran **10** as a diastereomeric mixture. Next, the base-induced elimination of HBr converted **10** to diene **11**, which underwent the Claisen rearrangement at 170 °C to give rise to heptenone **12** [17–21]. The more thermodynamically stable silyl enol ether **13** was regioselectively formed from **12** under Holton’s conditions [22], and DDQ-mediated oxidation of **13** resulted in the formation of α,β-unsaturated ketone **14**. Asymmetric reduction of ketone **14** was in turn realized by applying a stoichiometric amount of (*R*)-CBS and BH_3_·SMe_2_ to produce **15** (82% ee) [23]. The absolute configuration of C4 was determined as *S* by the modified Mosher method after acylation of **15** using (*R*)- and (*S*)-MTPACl [24]. Finally, the hydroxy group of **15** was protected as its TBS ether to afford **7**.

**Scheme 3:**
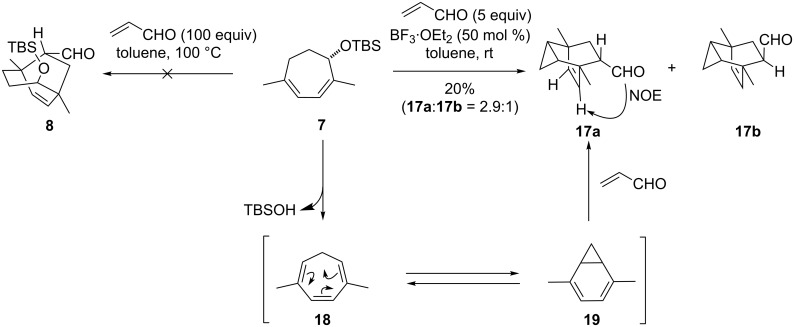
Generation of **17** through the 6π-electrocyclic reaction and the Diels–Alder reaction.

We then explored the Diels–Alder reaction between **7** and acrolein to construct the bicyclo[3.2.2]nonene structure (Scheme 3). The Diels–Alder reaction under thermal conditions (100 °C) induced the decomposition of diene **7**, and only the starting material was recovered after 10 h (26%). Because of the low reactivity of **7**, we applied a Lewis acid to facilitate the reaction. However, the reaction of **7** and acrolein in the presence of BF_3_·OEt_2_ (50 mol %) led to formation of the unexpected bicyclo[2.2.2]octene skeletons **17a** and **17b** as a 2.9:1 mixture. Under these conditions, BF_3_·OEt_2_-promoted elimination of the allylic siloxy group in **7** generated triene **18**, which then isomerized into **19** via a 6π-electrocyclic reaction. Diene **19**, which appeared to be more reactive than the original diene **7**, then underwent the Diels–Alder reaction to provide **17a** and **17b**.

Formation of **17a** and **17b** in Scheme 3 confirmed that selective activation of acrolein in the presence of the Lewis-basic allylic oxygen was a prerequisite to the successful formation of **8**. We anticipated that bulky trialkylsilyl triflates would function as such chemoselective Lewis acids, because the activation of the TBS-connected oxygen of **7** by the silyl triflate would cause highly unfavorable steric interactions. Indeed, the cycloaddition between **7** and acrolein proceeded even at −78 °C by the action of TMSOTf (50 mol %) in toluene to afford the bicyclo[3.2.2]nonene **8** and **20** along with a small amount of two other isomers **21ab** (Table 1, entry 1). Among the various silyl triflates used (Table 1, entries 1–3), TBSOTf was found to be superior to TMSOTf or TIPSOTf in terms of the combined yield. Alteration of the amount of TBSOTf from 50 mol % (Table 1, entry 2) to 200 mol % (Table 1, entry 4) and change of the solvent from toluene (Table 1, entry 4) to CH_2_Cl_2_ (Table 1, entry 5) increased the yield of the adducts. It is particularly worthy of note that the use of 2,6-di-*tert*-butylpyridine in combination with 200 mol % of TBSOTf effectively inhibited the Lewis-acid-promoted elimination of the C4-oxy group, and that the ratio of **8** to **20** was improved from 1.6:1 to 3.4:1 by replacement of the solvent (Table 1, entries 4 and 5). Thus, we developed an effective method for synthesis of the requisite stereoisomer **8** by applying a TBSOTf-promoted Diels–Alder reaction [25–26]. Most importantly, the C4-stereocenter behaved as the control element to introduce the two quaternary carbons (C1 and 5) and the C12-stereocenter.

**Table 1:** Optimization of the Diels–Alder reaction.^a^

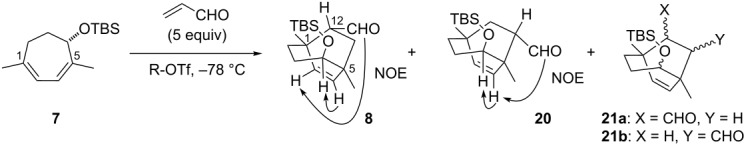				

entry	silyl triflate (R)	solvent	yield^b^	ratio^c^ (**8**/**20**/**21ab**)

1	TMS (50 mol %)	toluene	31%	1.9:1:0.6
2	TBS (50 mol %)	toluene	59%	1.8:1:0.5
3	TIPS (50 mol %)	toluene	47%	1.9:1:0.4
4^d^	TBS (200 mol %)	toluene	69%	1.6:1:0.4
5^d^	TBS (200 mol %)	CH_2_Cl_2_	88%	3.4:1:0.5

^a^Reaction was performed at 0.5 M. ^b^Combined yield of **8**, **20** and two other isomers **21a**/**21b**. ^c^Ratio was determined by ^1^H NMR analysis. ^d^2,6-Di-*tert*-butylpyridine (200 mol %) was added as a buffer.

**Scheme 4:**
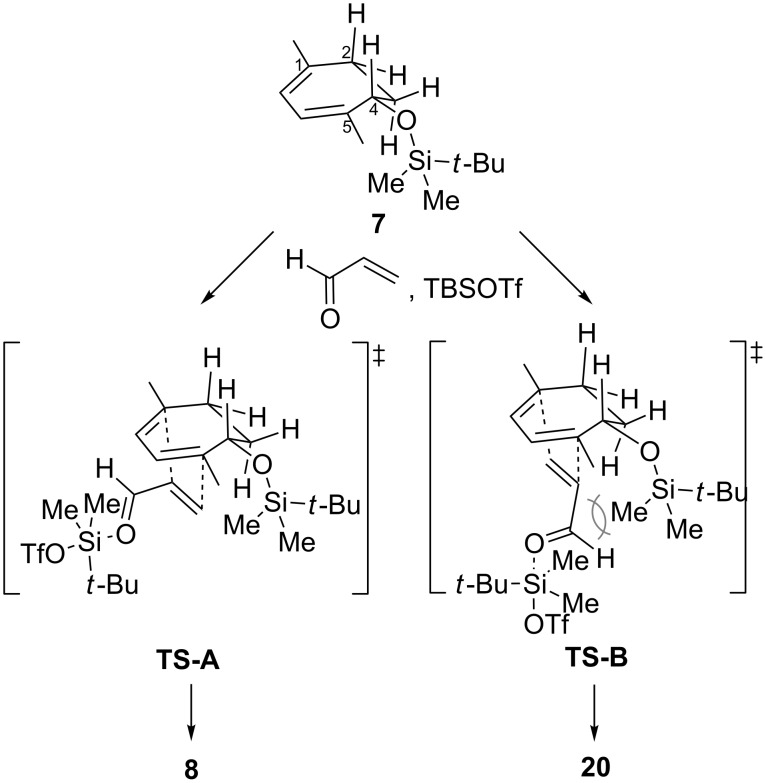
Rationale of the stereoselectivity of the Diels–Alder reaction.

The selective formation of **8** out of eight possible isomers is rationalized in Scheme 4. The *endo*-type transition states would be favored over their *exo*-type counterparts, and acrolein would approach from the bottom face of **7** to avoid steric interactions with the axially oriented C2- and C4-hydrogen atoms on the top face [27]. These considerations eliminate six out of the eight stereoisomeric transition states, and leave only **TS-A** and **TS-B**, which in fact correspond to the generated adducts **8** and **20**, respectively. **TS-A** would be preferred over **TS-B** due to the unfavorable interaction of the two proximal TBS groups in **TS-B**, allowing formation of **8** as the major compound.

**Scheme 5:**
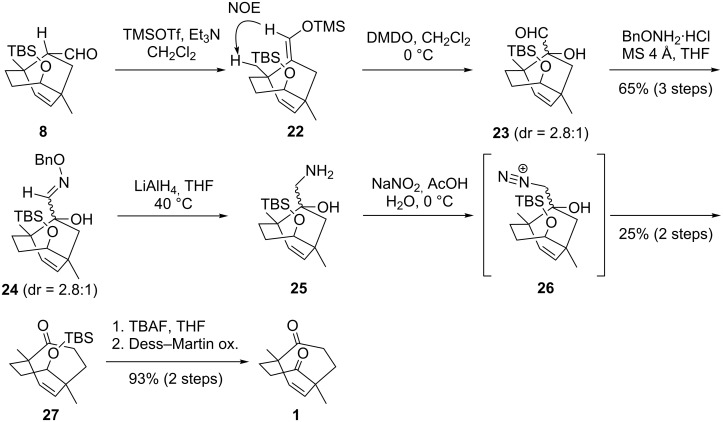
Synthesis of *C*_2_-symmetric **1**.

Having synthesized the optically active **8**, the next task was the preparation of *C*_2_-symmetric bicyclo[3.3.2]decene **1** from **8** (Scheme 5). The silyl enol ether formation of aldehyde **8** afforded **22** as a single stereoisomer, and the obtained **22** was oxidized with DMDO to provide α-hydroxy aldehyde **23** as a diastereomeric mixture (dr = 2.8:1). Compound **23** then reacted with benzyl hydroxylamine to produce oxime **24**, LiAlH_4_-treatment of which led to **25**. The regioselective ring expansion of seven-membered **25** was induced by treatment with NaNO_2_ in acetic acid [28–29], resulting in the formation of eight-membered **27** through the intermediary of **26**. Finally, the desilylation of **27** with TBAF and the subsequent oxidation of the resultant hydroxy group delivered the symmetric diketone **1** in optically active form.

## Conclusion

In summary, we developed a synthetic route to the optically active seven-membered **7** and established the TBSOTf-promoted stereoselective Diels–Alder reaction between **7** and acrolein to construct highly functionalized bicyclo[3.2.2]nonene **8** bearing two quaternary carbons. Seven additional transformations of **8**, including the ring expansion of the seven-membered ring to an eight-membered ring, delivered *C*_2_-symmetric bicyclo[3.3.2]decene **1**, which is the key intermediate in our synthetic studies of ryanodine.

## Experimental

**General:** All reactions sensitive to air or moisture were carried out under argon or nitrogen atmosphere in dry, freshly distilled solvents under anhydrous conditions, unless otherwise noted. All other reagents were used as supplied unless otherwise stated. Analytical thin-layer chromatography (TLC) was performed by using E. Merck Silica gel 60 F254 precoated plates. Flash column chromatography was performed by using 40–50 µm Silica Gel 60N (Kanto Chemical Co., Inc.), 40–63 µm Silicagel 60 (Merck) or 32–53 µm Silica-gel BW-300 (Fuji Silysia Chemical Ltd.). Melting points were measured on a Yanaco MP-J3 micro melting-point apparatus and are uncorrected. Optical rotations were recorded on a JASCO DIP-1000 Digital Polarimeter. Infrared (IR) spectra were recorded on a JASCO FT/IR-4100 spectrometer. ^1^H and ^13^C NMR spectra were recorded on JEOL JNM-ECX-500, JNM-ECA-500, or JNM-ECS-400 spectrometers. Chemical shifts were reported in parts per million (ppm) on the δ scale relative to CHCl_3_ (δ 7.26 for ^1^H NMR), CDCl_3_ (δ 77.0 for ^13^C NMR), C_6_D_5_H (δ 7.16 for ^1^H NMR), C_6_D_6_ (δ 128.0 for ^13^C NMR), CO(CD_3_)(CD_2_H) (δ 2.05 for ^1^H NMR) as internal references. Signal patterns are indicated as s, singlet; d, doublet; t, triplet; q, quartet, m, multiplet. The numbering of the compounds corresponds to that of the natural products. High-resolution mass spectra were measured on Bruker microTOFII.

**TMS-enol ether 13:** Methylmagnesium bromide (3.0 M in Et_2_O, 8.5 mL, 26 mmol) was added to a solution of iPr_2_NH (3.9 mL, 28 mmol) in Et_2_O (170 mL) at 0 °C. The mixture was stirred at room temperature for 19 h, and cooled to −78 °C. Then, a solution of **12** [3.5 g as a 5.7:1 mixture of **12** (23 mmol) and Et_2_O] in Et_2_O (60 mL), TMSCl (9.1 mL, 72 mmol), Et_3_N (11.3 mL, 81 mmol), and HMPA (2.0 mL, 11 mmol) were successively added to the mixture. The reaction mixture was stirred at room temperature for 18 h and cooled to 0 °C. Phosphate buffer (pH 7, 100 mL) was added, and the resultant solution was extracted with Et_2_O (150 mL × 3). The combined organic layers were washed with H_2_O (30 mL) and brine (200 mL), dried over Na_2_SO_4_, filtered, and concentrated. The residue was purified by flash column chromatography (silica gel 200 g, pentane only) to afford silyl enol ether **13** (3.3 g, 16 mmol) and its regioisomer (344 mg, 1.64 mmol) in 70% and 7% yield, respectively. The synthesized silyl enol ether **13** was immediately subjected to the next reaction: colorless oil; ^1^H NMR (500 MHz, C_6_D_6_) δ 0.17 (9H, s, C*H*_3_ of TMS), 1.61 (3H, d, *J* = 1.2 Hz, H17), 1.73 (3H, s, H20), 2.05 (2H, t, *J* = 6.3 Hz, H2ab), 2.31–2.35 (2H, m, H3ab), 2.60 (2H, d, *J* = 5.7 Hz, H14ab), 5.53 (1H, tq, *J* = 5.7, 1.2 Hz, H15); ^13^C NMR (125 MHz, C_6_D_6_) δ 1.2, 19.3, 25.8, 30.9, 31.7, 32.8, 114.7, 123.3, 137.4, 146.4. The regioisomer of **13**: colorless oil; ^1^H NMR (400 MHz, C_6_D_6_) δ 0.18 (9H, s, C*H*_3_ of TMS), 1.19 (3H, d, *J* = 6.9 Hz, H20), 1.67 (3H, br s, H17), 2.00 (1H, ddd, *J* = 14.6, 7.3, 7.3 Hz, H14a), 2.23–2.27 (1H, m, H14b), 2.32–2.40 (1H, m, H5), 2.46 (1H, dd, *J* = 17.9, 6.4 Hz, H2a), 2.62 (1H, dd, *J* = 17.9, 5.5 Hz, H2b), 4.95 (1H, dd, *J* = 6.4, 5.5 Hz, H3), 5.49 (1H, br ddq, *J* = 7.3, 7.3, 1.4 Hz, H15).

**Diene 14:** DDQ (7.1 g, 31 mmol) was added to a solution of **13** (3.3 g 16 mmol) and 2,6-lutidine (5.4 mL, 46 mmol) in benzene (30 mL) at 0 °C. The reaction mixture was stirred at room temperature for 20 min and filtered through a short column of Al_2_O_3_ with Et_2_O. The filtrate was concentrated. The residue was purified by flash column chromatography (silica gel 120 g, pentane/Et_2_O 10:1 to 5:1) to afford diene **14** (1.5 g, 11 mmol) in 69% yield: yellow oil; IR (neat) ν_max_: 2949, 1656, 1595, 1431, 1377 cm^−1^; ^1^H NMR (500 MHz, CDCl_3_) δ 1.92 (3H, s, H20), 1.98 (3H, s, H17), 2.29 (2H, t, *J* = 6.3 Hz, H2ab), 2.64 (2H, t, *J* = 6.3 Hz, H3ab), 5.80 (1H, d, *J* = 8.0 Hz, H15), 6.51 (1H, d, *J* = 8.0 Hz, H14); ^13^C NMR (100 MHz, CDCl_3_) δ 20.4, 26.5, 28.1, 41.7, 122.4, 136.0, 137.0, 150.0, 201.3; HRMS–ESI (*m*/*z*): [M + Na]^+^ calcd for C_9_H_12_ONa, 159.0780; found, 159.0778.

**Diene 15:** BH_3_·SMe_2_ (220 μL, 2.3 mmol) was added to a solution of (*R*)-2-Me-CBS-oxazaborolidine (1.0 M in toluene, 2.3 mL, 2.3 mmol) in THF (6 mL) at 0 °C. The solution was stirred for 30 min at 0 °C and cooled to −78 °C. Then a solution of diene **14** (265 mg, 1.94 mmol) in THF (3 mL) was added at −78 °C. The reaction mixture was warmed to 0 °C over 15 min, and H_2_O (10 mL) was added. The resultant solution was extracted with Et_2_O (6 mL × 3). The combined organic layers were washed with brine (5 mL), dried over Na_2_SO_4_, filtered and concentrated. The residue was purified by flash column chromatography (silica gel 5 g, pentane/Et_2_O 20:1 to 5:1) to afford **15** (261 mg**,** 1.89 mmol) in 97% yield. The enantiopurity of **15** was determined as 82% ee by comparison of the integrations of the H14 peak at 5.78 and 5.72 ppm on ^1^H NMR after the esterification with (*S*)-MTPACl; yellow oil; [α]_D_^20^ −402 (*c* 0.995, CHCl_3_); IR (neat) ν_max_: 3336, 2965, 2920, 2880, 1434, 1056, 1013 cm^−1^; ^1^H NMR (400 MHz, CDCl_3_) δ 1.54 (1H, br s, O*H*), 1.75 (1H, dddd, *J* = 13.7, 11.9, 2.8, 2.8 Hz, H3a), 1.85 (3H, s, H17 or 20), 1.95 (3H, s, H17 or 20), 2.00–2.15 (2H, m, H2a and 3b), 2.32–2.39 (1H, br dd, *J* = 16.5, 11.9 Hz, H2b), 4.20 (1H, m, H4), 5.52 (1H, br d, *J* = 7.8 Hz, H14 or 15), 5.61 (1H, d, *J* = 7.8 Hz, H14 or 15); ^13^C NMR (125 MHz, C_6_D_6_) δ 24.0, 26.6, 29.2, 33.2, 72.4, 120.7, 122.6, 140.2, 142.9; HRMS–ESI (*m*/*z*): [M + Na]^+^ calcd for C_9_H_14_ONa, 161.0937; found, 161.0934.

**Diene 7:** The mixture of **15** (259 mg, 1.88 mmol), imidazole (300 mg, 4.41 mmol) and TBSCl (330 mg, 2.19 mmol) in DMF (6.0 mL) was stirred for 8.5 h at room temperature and cooled to 0 °C. H_2_O (10 mL) was added to the reaction mixture, and the resultant solution was stirred for 30 min. The mixture was extracted with EtOAc (6 mL × 3). The combined organic layers were dried over Na_2_SO_4_, filtered and concentrated. The residue was purified by flash column chromatography (silica gel 5 g, hexane/EtOAc 1:0 to 100:1) to afford **7** (404 mg, 1.60 mmol) in 85% yield; colorless oil; [α]_D_^20^ −247 (*c* 1.04, CHCl_3_); IR (neat) ν_max_: 2956, 2928, 2856, 1472, 1436, 1253 cm^−1^; ^1^H NMR (400 MHz, CDCl_3_) δ 0.085 (3H, s, C*H*_3_ of TBS), 0.093 (3H, s, C*H*_3_ of TBS), 0.91 (9H, s, *t*-Bu of TBS), 1.75 (1H, dddd, *J* = 13.3, 10.5, 2.3, 2.3 Hz, H3a), 1.83 (3H, s, H17), 1.85 (3H, s, H20), 1.92–2.00 (1H, dddd, *J* = 13.3, 8.7, 6.4, 2.3 Hz, H3b), 2.04 (1H, ddd, *J* = 16.5, 8.7, 2.3 Hz, H2a), 2.40 (1H, br dd, *J* = 16.5, 10.5 Hz, H2b), 4.22 (1H, br d, *J* = 6.4 Hz, H4), 5.50 (1H, d, *J* = 7.8 Hz, H15), 5.54, (1H, d, *J* = 7.8 Hz, H14); ^13^C NMR (100 MHz, CDCl_3_) δ −4.3, −4.1, 18.5, 24.0, 26.2, 26.9, 30.0, 34.9, 73.4, 120.5, 121.5, 141.4, 143.2; HRMS–ESI (*m*/*z*): [M + Na]^+^ calcd for C_15_H_28_OSiNa, 275.1802; found, 275.1801.

**Cycloadduct 8 and 20:** TBSOTf (530 μL, 2.3 mmol) was added to a solution of **7** (297 mg, 1.18 mmol), acrolein (390 μL, 5.8 mmol) and 2,6-di-*tert*-butylpyridine (530 μL, 2.3 mmol) in CH_2_Cl_2_ (2.4 mL) at −78 °C. The reaction mixture was stirred for 15 min at −78 °C, and then saturated aqueous NaHCO_3_ (3 mL) was added. The resultant mixture was extracted with EtOAc (5 mL × 3). The combined organic layers were dried over Na_2_SO_4_, filtered and concentrated. The residue was purified by flash column chromatography (silica gel 12 g, hexane/CH_2_Cl_2_ 4:1 to 3:1) to afford pure **20** (23 mg, 75 μmol), a mixture of **20**, **21a**, **21b** and **8** (215 mg, 0.67 mmol), and pure **8** (87 mg, 0.29 mmol) in 88% combined yield. **8**: colorless oil; [α]_D_^20^ +25.6 (*c* 1.13, CHCl_3_); IR (neat) ν_max_: 2954, 2930, 1724, 1253, 1070 cm^−1^; ^1^H NMR (400 MHz, CDCl_3_) δ 0.02 (6H, s, C*H*_3_ of TBS × 2), 0.88 (9H, s, *t*-Bu of TBS), 1.02 (3H, s, H17 or 20), 1.03 (3H, s, H17 or 20), 1.21 (1H, dd, *J* = 14.6, 4.1 Hz, H6a), 1.42 (1H, ddd, *J* = 13.7, 5.5, 5.0 Hz, H2a), 1.56 (1H, ddd, *J* = 13.7, 11.5, 5.0 Hz, H2b), 1.72 (1H, dddd, *J* = 14.6, 11.5, 9.2, 5.5 Hz, H3a), 1.84 (1H, dddd, *J* = 14.6, 5.0, 5.0, 5.0 Hz, H3b), 2.34 (1H, dd, *J* = 14.6, 9.6 Hz, H6b), 2.57 (1H, ddd, *J* = 9.6, 5.5, 4.1 Hz, H12), 3.38 (1H, dd, *J* = 9.2, 5.0 Hz, H4), 5.76 (1H, d, *J* = 9.2 Hz, H14), 5.81 (1H, d, *J* = 9.2 Hz, H15), 9.29 (1H, d, *J* = 5.5 Hz, C*H*O); ^13^C NMR (100 MHz, CDCl_3_) δ −4.8, −4.2, 18.1, 25.9, 26.7, 27.0, 29.2, 34.5, 35.3, 40.2, 40.4, 56.5, 71.7, 138.1, 138.3, 204.4; HRMS–ESI (*m*/*z*): [M + Na]^+^ calcd for C_18_H_32_O_2_SiNa, 331.2064; found, 331.2063. **20**: IR (neat) ν_max_: 2954, 2930, 2856, 1724, 1253, 1070 cm^−1^; ^1^H NMR (400 MHz, CDCl_3_) δ 0.02 (6H, s, C*H*_3_ of TBS × 2), 0.88 (9H, s, *t*-Bu of TBS), 1.02 (3H, s, C*H*_3_CCHCHO), 1.06 (3H, s, C*H*_3_CCH_2_), 1.34 (1H, ddd, *J* = 13.7, 6.0, 3.7 Hz, CC*H*_A_H_B_CH_2_), 1.52–1.59 (2H, m, CCH_A_*H*_B_CH_2_ and CC*H*_A_H_B_CHCHO), 1.70–1.87 (2H, m, CCH_2_C*H*_2_CH(OTBS)), 1.91 (1H, dd, *J* = 14.7, 10.1 Hz, CCH_A_*H*_B_CHCHO), 2.97 (1H, ddd, *J* = 10.1, 5.0, 5.0 Hz, C*H*CHO), 3.32 (1H, dd, *J* = 9.2, 5.5 Hz, C*H*(OTBS)), 5.55 (1H, d, *J* = 9.2 Hz, CC*H*=CHCCH_2_), 5.97 (1H, d, *J* = 9.2 Hz, CCH=C*H*CCH_2_), 9.37 (1H, d, *J* = 5.0 Hz, C*H*O); ^13^C NMR (125 MHz, CDCl_3_) δ −4.8, −4.2, 18.1, 24.1, 25.9, 29.7, 33.8, 34.1, 34.6, 38.0, 41.8, 50.6, 73.5, 134.1, 142.4, 204.3; HRMS–ESI (*m*/*z*): [M + Na]^+^ calcd for C_18_H_32_O_2_SiNa, 331.2064; found, 331.2057.

**Oxime 24:** TMSOTf (220 μL, 1.2 mmol) was added to a solution of **8** (122 mg, 0.395 mmol) and Et_3_N (330 μL, 2.4 mmol) in CH_2_Cl_2_ (2.0 mL) at 0 °C. The reaction mixture was stirred at room temperature for 15 h and cooled to 0 °C. Phosphate buffer (pH 7, 5 mL) was added, and the resultant mixture was extracted with EtOAc (6 mL × 3). The combined organic layers were dried over Na_2_SO_4_, filtered and concentrated. The residue was passed through a short column (silica gel 100 mg, hexane/EtOAc 2:1) to afford the crude TMS enol ether **22**, which was used in the next reaction without further purification. DMDO (58 mM in acetone, 6.8 mL, 0.39 mmol) was added to a solution of the above crude TMS enol ether **22** in CH_2_Cl_2_ (1.0 mL). The reaction mixture was stirred for 10 min at 0 °C, and then isoprene (39 μL, 0.39 mmol) was added. The resultant solution was concentrated. The residue was roughly purified by flash column chromatography (silica gel 24 g, hexane/EtOAc 1:0 to 10:1) to afford hydroxy aldehyde **23** (93 mg) as a diastereomixture (2.8:1), which was used in the next reaction without further purification. A mixture of the above crude **23**, BnONH_2_·HCl (135 mg, 0.846 mmol), and MS4Å (93 mg) in THF (3.0 mL) was stirred at room temperature for 22 h. The reaction mixture was filtered through a pad of Celite with EtOAc (15 mL), and the filtrate was concentrated. The residue was purified by flash column chromatography (silica gel 15g, hexane/toluene 2:1 to 0:1 then hexane/CH_2_Cl_2_ 1:1 to 1:3) to afford oxime **24a** (81 mg, 0.19 mmol) and **24b** (29 mg, 67 μmol) in 48% and 17% yield, respectively, over three steps. **24a**: colorless oil: [α]_D_^20^ +60 (*c* 0.73, CHCl_3_); IR (neat) ν_max_: 3522, 2954, 2929, 2856, 1455, 1363, 1254, 1067 cm^−1^; ^1^H NMR (500 MHz, CDCl_3_) δ 0.006 (3H, s, C*H*_3_ of TBS), 0.012 (3H, s, C*H*_3_ of TBS), 0.88 (9H, s, *t*-Bu of TBS), 0.98 (3H, s, H17 or 20), 1.02 (3H, s, H17 or 20), 1.33 (1H, d, *J* = 16.1 Hz, H6a), 1.36–1.57 (3H, m, H2ab and 3a), 1.85 (1H, dddd, *J* = 14.9, 5.8, 5.8, 5.8 Hz, H3b), 2.81 (1H, d, *J* = 16.1 Hz, H6b), 2.94 (1H, s, O*H*), 3.40 (1H, dd, *J* = 6.3, 5.8 Hz, H4), 5.13 (2H, s, OC*H*_2_Ph), 5.73 (1H, d, *J* = 9.2 Hz, H14 or 15), 5.79 (1H, d, *J* = 9.2 Hz, H14 or 15), 7.30–7.39 (5H, m, aromatic), 7.71 (1H, s, C*H*NOBn); ^13^C NMR (125 MHz, CDCl_3_) δ −4.8, −4.3, 18.1, 23.3, 25.9, 27.0, 32.6, 33.9, 39.7, 42.6, 42.9, 71.0, 76.3, 78.0, 128.0, 128.42, 128.44, 137.1, 137.3, 138.4, 152.8; HRMS–ESI (*m*/*z*): [M + Na]^+^ calcd for C_25_H_39_NO_3_SiNa, 452.2591; found, 452.2589. **24b**: IR (neat) ν_max_: 3525, 3025, 2955, 2928, 2856 cm^−1^; ^1^H NMR (400 MHz, CDCl_3_) δ 0.01 (3H, s, C*H*_3_ of TBS), 0.02 (3H, s, C*H*_3_ of TBS), 0.83 (3H, s, H17), 0.88 (9H, s, *t*-Bu of TBS), 0.95 (3H, s, H20), 1.33–1.42 (1H, m, H2a), 1.50 (1H, d, *J* = 15.1 Hz, H6a), 1.80–1.88 (2H, m, H2b and 3a), 1.96–2.07 (1H, m, H3b), 2.44 (1H, d, *J* = 15.1 Hz, H6b), 3.42 (1H, dd, *J* = 8.2, 6.0 Hz, H4), 3.48 (1H, s, O*H*), 5.06 (2H, s, OC*H*_2_Ph), 5.62 (1H, d, *J* = 9.6 Hz, H14), 5.65 (1H, d, *J* = 9.6 Hz, H15), 7.27–7.35 (5H, m, aromatic), 7.39 (1H, s, C*H*NOBn); ^13^C NMR (100 MHz, CDCl_3_) δ −4.8, −4.1, 18.1, 23.5, 25.9, 26.7, 34.3, 34.9, 41.4, 41.5, 42.3, 71.6, 76.2, 78.1, 127.9, 128.3, 128.4, 137.2, 137.6, 138.3, 156.2; HRMS–ESI (*m*/*z*): [M + Na]^+^ calcd for C_25_H_39_NO_3_SiNa, 452.2591; found, 452.2580.

**Ketone 27:** LiAlH_4_ (2.0 M in THF, 380 μL, 0.76 mmol) was added to a solution of oxime **24** (110 mg, 0.256 mmol, a 2.8:1 diastereomixture of **24a** and **24b**) in THF at 0 °C. The reaction mixture was stirred at 0 °C for 10 min, at room temperature for 2.5 h, and at 40 °C for 2 h. After additional LiAlH_4_ (2.0 M in THF, 380 μL, 0.76 mmol) was added, the reaction mixture was stirred at 50 °C for a further 3.5 h. LiAlH_4_ (2.0 M in THF, 380 μL, 0.76 mmol) was added again, and the reaction mixture was stirred for a further 1 h. After the reaction mixture was cooled to 0 °C, H_2_O (87 μL) was added. The resultant solution was stirred for 1 h at room temperature, and then 15% aqueous NaOH (87 μL) and H_2_O (260 μL) were added. The solution was stirred for 9 h. The resultant mixture was filtered through a pad of Celite with THF (30 mL), and the filtrate was concentrated to afford amino alcohol **25**, which was used in the next reaction without further purification. A solution of NaNO_2_ (141 mg, 2.04 mmol) in H_2_O (1.3 mL) was added to a solution of the above crude amino alcohol **25** in H_2_O (3.8 mL) and AcOH (1.0 mL) at 0 °C. The reaction mixture was stirred at 0 °C for 3 h, and then saturated aqueous NaHCO_3_ (30 mL) was added. The resultant mixture was extracted with EtOAc (8 mL × 4). The combined organic layers were dried over Na_2_SO_4_, filtered and concentrated. The residue was purified by flash column chromatography (silica gel 18 g, hexane/CH_2_Cl_2_ 3:1 to 0:1) to afford ketone **27** (20 mg, 65 μmol) in 25% yield over two steps: colorless oil; [α]_D_^20^ +134 (*c* 0.165, CHCl_3_); IR (neat) ν_max_: 2954, 2929, 2856, 1703, 1462, 1063 cm^−1^; ^1^H NMR (500 MHz, CDCl_3_) δ 0.01 (6H, s, C*H*_3_ of TBS × 2), 0.86 (9H, s, *t*-Bu of TBS), 1.11 (3H, s, H17 or 20), 1.12 (3H, s, H17 or 20), 1.41 (1H, ddd, *J* = 14.9, 8.1, 2.3 Hz, H6a), 1.52–1.72 (3H, m, H2ab and 3a), 1.75–1.81 (1H, m, H3b), 2.25 (1H, ddd, *J* = 12.6, 5.2, 2.3 Hz, H11a), 2.34 (1H, ddd, *J* = 14.9, 12.6, 5.2 Hz, H6b), 2.93 (1H, ddd, *J* = 12.6, 12.6, 8.1 Hz, H11b), 3.40 (1H, dd, *J* = 10.3, 4.6 Hz, H4), 5.56 (1H, d, *J* = 10.9 Hz, H14), 5.73 (1H, d, *J* = 10.9 Hz, H15); ^13^C NMR (125 MHz, CDCl_3_) δ −4.7, −4.2, 18.0, 25.9, 26.4, 29.0, 31.2, 34.6, 40.0, 42.2, 42.4, 50.3, 73.7, 137.6, 139.4, 218.5; HRMS–ESI (*m*/*z*): [M + Na]^+^ calcd for C_18_H_32_O_2_SiNa, 331.2064; found, 331.2078.

***C*_2_-symmetric diketone 1:** TBAF (1.0 M in THF, 130 μL, 0.13 mmol) was added to a solution of **27** (13 mg, 42 μmol) in THF (0.9 mL) at room temperature. The reaction mixture was stirred for 1.5 h at room temperature and at 40 °C for 1.5 h. TBAF (1.0 M in THF, 83 μL, 83 μmol) was added again to the reaction mixture, and the reaction mixture was stirred at 40 °C for a further 16.5 h. The reaction mixture was cooled to room temperature, and passed through a short column of silica gel with Et_2_O. The filtrate was concentrated to afford crude alcohol, which was used in the next reaction without further purification. A mixture of the above crude alcohol, NaHCO_3_ (33 mg, 0.39 mmol), and Dess–Martin periodinane (53 mg, 0.13 mmol) in CH_2_Cl_2_ (0.8 mL) was stirred at room temperature for 2.5 h, and then saturated aqueous Na_2_S_2_O_3_ (3 mL) was added. The resultant mixture was extracted with Et_2_O (4 mL × 3). The combined organic layers were washed with brine (5 mL), dried over Na_2_SO_4_, filtered and concentrated. The residue was purified by flash column chromatography (silica gel 1 g, pentane/Et_2_O 5:1 to 2:1) to afford *C*_2_-symmetric diketone **1** (7.5 mg, 39 μmol) in 93% yield over two steps: crystal; m.p. 55 °C; [α]_D_^20^ +260 (*c* 0.37, CHCl_3_). Other analytical data of **1** were identical to those reported by our group previously [11].

## Supporting Information

File 1 Experimental procedures and NMR spectra of all newly synthesized compounds.
