# Supplementary material for: Asymmetric synthesis of a highly functionalized bicyclo[3.2.2]nonene derivative
Source: Beilstein J Org Chem. 2013 Apr 4;9:655–63. doi: 10.3762/bjoc.9.74 (PMC3629056; doi:10.3762/bjoc.9.74)
Supplement: File 1 — Experimental procedures and NMR spectra of all newly synthesized compounds. [file Beilstein_J_Org_Chem-09-655-s001.pdf]

Supporting Information

for

Asymmetric synthesis of a highly functionalized bicyclo[3.2.2]nonene derivative

Toshiki Tabuchi, Daisuke Urabe and Masayuki Inoue*

Address: Graduate School of Pharmaceutical Sciences, The University of Tokyo, Hongo, Bunkyo-ku, Tokyo 133-0033 Japan

Email: Masayuki Inoue - inoue@mol.f.u-tokyo.ac.jp

* Corresponding author

Experimental procedures and NMR spectra of all newly synthesized compounds

Contents

Experimental procedure for syntheses of compound **9**, **10**, **12**, **16**, and **17a** S2

^{1}H and ^{13}C NMR spectra of newly synthesized compounds S5

Allyl alcohol 9: Crotylchloride (10 mL, 0.10 mol) was added to a solution of acetylacetone (21 mL, 0.20 mol) and K_2CO_3 (18 g, 0.13 mol) in EtOH (200 mL) at 70 °C. The resulting solution was stirred at 70 °C for 22 h, and cooled to room temperature. H_2O (100 mL) was added to the mixture. The resultant mixture was extracted with Et_2O (30 mL × 3), and the organic layers were washed with H_2O (200 mL). The aqueous layer was re-extracted with Et_2O (150 mL × 3) and pentane (150 mL × 3). The combined organic layers were washed with H_2O (100 mL × 3), dried over Na_2SO_4, filtered and concentrated to afford the crude 2-hepten-6-one. A solution of vinylmagnesium bromide (1.0 M THF solution, 111 mL, 111 mmol) was added to the solution of the above crude 2-hepten-6-one in CH_2Cl_2 (180 mL) at 0 °C. The reaction mixture was stirred at 0 °C for 20 min, and then saturated aqueous NH_4Cl (40 mL) was added. The resultant solution was extracted with Et_2O (30 mL × 3). The combined organic layers were washed with brine (30 mL), dried over Na_2SO_4, filtered and concentrated. The residue was purified by flash column chromatography (silica gel 250 g, pentane/Et_2O 20:1 to 1:1) to afford allyl alcohol **9** [11.7 g as a 1.8:1 mixture of **9** (65 mmol, *E*/*Z* = 4/1) and Et_2O] in 65% calculated yield from ^{1}H NMR over 2 steps: yellow oil. Characterization data were identical with those previously reported (Cane, D. E.; Thomas, P. J. *J. Am. Chem. Soc.* **1984,** *106*, 5295–5303).

Bromide 10: NBS (11.5 g, 64.5 mmol) was added to a solution of **9** [11.7 g as a 1.8:1 mixture of **9** (65 mmol, *E*/*Z* = 4/1) and Et_2O] in CCl_4 (110 mL) at 0 °C. The reaction mixture was stirred at room temperature for 23 h, and at 30 °C for 18 h. The mixture was directly subjected to a flash column chromatography (silica gel 400 g, pentane/Et_2O 100:1 to 20:1) to give **10** [15.4 g as a 1.1:1 mixture of **10** (53.4 mmol) and Et_2O] as a diastereomixture (13:4:3:1), which was used in the next reaction without further purification: ^{1}H NMR of the major isomer of **10** (400 MHz, $CDCl_3$) δ 1.30 (3H, s, CH_3C), 1.64–2.2 (4H, m, CCH_2CH_2), 1.76 (3H, d, *J* = 6.8 Hz, CH_3CHBr), 3.96–4.06 (2H, m, C*H*Br, C*H*O), 5.00 (1H, dd, *J* = 11.0, 1.4 Hz, CH_AH_B=CH), 5.19 (1H, dd, *J* = 17.4, 1.4 Hz, CH_AH_B=CH), 5.92 (1H, dd, *J* = 17.4, 11.0 Hz, CH_2=C*H*).

Cycloheptenone 12: The mixture of the above diastereomixture of **10** [15.4 g as a 1.1:1 mixture of **10** (53.4 mmol) and Et_2O] and DBU (16.0 mL, 107 mmol) in 2,4,6-collidine (90 mL) was stirred at 110 °C for 3.5 h, and 170 °C for 16 h. After being cooled to room temperature, the reaction mixture was poured into an ice-cold 10% aqueous HCl (100 mL). The resulting mixture was extracted with Et_2O (100 mL × 3). The combined organic layers were washed with 10% aqueous HCl (100 mL × 2), saturated aqueous $NaHCO_3$ (100 mL) and brine (100 mL), dried over Na_2SO_4, filtered and concentrated. The residue was purified by flash column chromatography (silica gel 250 g, pentane/Et_2O 20:1 to 1:1) to afford cycloheptenone **12** [10.6 g as a 0.63:1 mixture of **12** (41 mmol) and Et_2O] in 63% calculated yield from ^{1}H NMR over 2 steps. For a characterization of **12**, Et_2O was completely removed from the mixture: red oil; IR (neat) ν_{max}: 2967, 2930, 1706, 1450 cm^{-1}; ^{1}H NMR (400 MHz, $CDCl_3$) δ 1.07 (3H, d, *J* = 6.8 Hz, H20), 1.73 (3H, s, H17), 2.08–2.17 (2H, m, H2a and 14a), 2.21–2.26 (1H, m, H14b), 2.40–2.49 (1H, m, H2b), 2.51 (1H, ddd, *J* = 14.2, 7.3, 3.6 Hz, H3a), 2.73 (1H, ddd, *J* = 14.2, 11.0, 4.1 Hz, H3b), 2.80 (1H, dqd, *J* = 11.0, 6.8, 4.1 Hz, H5), 5.51 (1H, m, H15); ^{13}C NMR (100 MHz, $CDCl_3$) δ 16.3, 25.8, 29.5, 32.2, 40.8, 45.9, 122.7, 137.2, 214.9; HRMS–ESI (*m*/*z*): $[M + Na]^+$ calcd for $C_9H_{14}ONa$, 161.0937; found, 161.0943.

MTPA ester 16: DMAP in CH_2Cl_2 (82 mM, 20 µL, 1.7 µmol) was added to a solution of **15** (2.3 mg, 17 µmol), Et_3N (18 µL, 0.13 mmol), and (*R*)-MTPACl (12 µL, 64 µmol) in CH_2Cl_2 (0.83 mL). The reaction mixture was stirred for 15 h at room temperature, and Et_3N (18 µL, 0.13 mmol) and (*R*)-MTPACl (12 µL, 64 µmol) were added. After being stirred for 8 h, the reaction mixture was quenched with H_2O (2 mL). The resultant solution was extracted with CH_2Cl_2 (3 mL × 4). The combined organic layers were dried over Na_2SO_4, filtered and concentrated to afford MTPA ester **16a**. According to this procedure, MTPA ester **16b** was synthesized from **15** (2.8 mg, 20 µmol) by using (*S*)-MTPACl (30 µL, 160 µmol), Et_3N (44 µL, 310 µmol), DMAP (82 mM, 25 µL, 2.1 µmol) in CH_2Cl_2 (1 mL). **16a**: ^{1}H NMR (400 MHz, acetone-d_6) δ 1.77 (3H, s, H17), 1.83 (1H, dddd, *J* = 14.6, 11.4, 2.8, 2.3 Hz, H3a), 1.87 (3H, s, H20), 2.02–2.16 (3H, m, H2ab and 3a), 3.59 (3H, br s, OCH_3), 5.54 (1H, d, *J* = 7.8 Hz, H15), 5.63 (1H, br d, *J* = 5.0 Hz, H4), 5.78 (1H, d, *J* = 7.8 Hz, H14), 7.46–7.57 (5H, m,

aromatic). **16b**: ^{1}H NMR (400 MHz, acetone-d_6) δ 1.66 (3H, s, H20), 1.83 (3H, s, H17), 1.89–1.97 (1H, m, H3a), 2.17 (2H, m, H2a and 3b), 2.32–2.38 (1H, m, H2b), 3.59 (3H, br s, OCH_3), 5.54 (1H, d, J = 7.8 Hz, H15), 5.66 (1H, br s, H4), 5.72 (1H, d, J = 7.8 Hz, H14), 7.46–7.59 (5H, m, aromatic).

Cyclopropane 17a: $BF_3{\cdot}OEt_2$ (5.0 µL, 40 µmol) was added to a solution of **7** (18 mg, 71 µmol) and acrolein (24 µL, 0.36 mmol) in toluene (0.14 mL) at room temperature. The reaction mixture was stirred for 20 min, and then quenched with saturated aqueous $NaHCO_3$ (1 mL). The resultant mixture was extracted with CH_2Cl_2 (2 mL × 4). The combined organic layers were dried over Na_2SO_4, filtered and concentrated. The residue was purified by flash column chromatography (silica gel 0.7 g, pentane/Et_2O 1:0 to 5:1) to afford impure cyclopropane **17ab**. The material was further purified by preparative-TLC (hexane/CH_2Cl_2 6:1) to afford cyclopropane **17ab** as a 2.9:1 diastereomixture (2.4 mg**,** 14 µmol) in 20% combined yield. The diasteromixture was further purified by preparative-TLC (hexane/CH_2Cl_2 6:1) to afford pure cyclopropane **17a** (1 mg) for the characterization: colorless oil; ^{1}H NMR (400 MHz, $CDCl_3$) δ 0.05–0.08 (2H, m, CHCH_2CH), 0.71–0.75 (2H, m, CHCH$_2$CH), 1.28 (3H, s, H17 or 20), 1.32 (3H, s, H17 or 20), 1.41 (1H, dd, J = 12.8, 4.1 Hz, CCH_AH$_B$CHCHO), 1.71 (1H, dd, J = 12.8, 9.6 Hz, CCH$_A$$H_B$CHCHO), 2.42 (1H, ddd, J = 9.6, 4.6, 4.1 Hz, CHCHO), 5.43 (1H, d, J = 8.2 Hz, CH=CHCCHCHO), 5.61 (1H, d, J = 8.2 Hz, CH=CHCCHCHO), 9.29 (1H, d, J = 4.6 Hz, CHO); ^{13}C NMR (100 MHz, $CDCl_3$) δ 1.2, 17.1, 17.4, 22.6, 24.1, 35.6, 36.7, 38.5, 59.1, 130.2, 135.0, 205.5; HRMS–ESI (m/z): $[M + Na]^+$ calcd for $C_{12}H_{16}ONa$, 199.1093; found, 199.1092.

Cycloheptenone 12

G:\PHannou_inoue\Tabuchi Toshiki\ryanodine\DATA\chiral-intermediate\7memberedring\7memberering without side chain(1-99).als

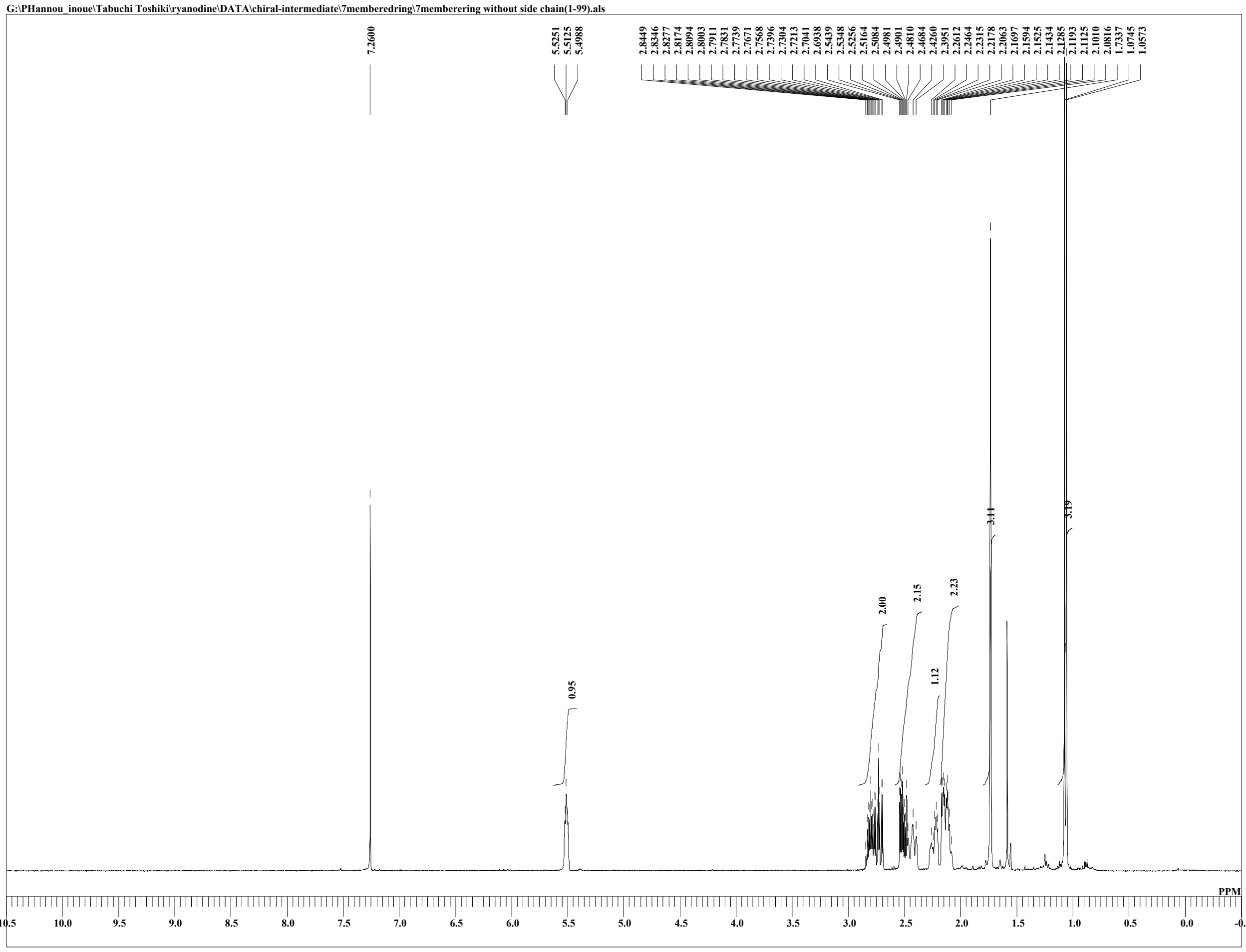

DFILE	7memberering without side chain
COMNT	TT1-090609-1-99-f10-16
DATIM	09-06-2009 12:23:38
MENUF	
OBNUC	1H
OFR	395.88 MHz
OBFRQ	395.88 MHz
OBSET	6.28 KHz
OBFIN	0.87 Hz
PW1	6.50 usec
DEADT	0.00 usec
PREDL	0.00000 msec
IWT	1.0000 sec
POINT	13107
SPO	13107
TIMES	16
DUMMY	1
FREQU	5938.15 Hz
FLT	30000 Hz
DELAY	16.68 usec
ACQTM	2.2073 sec
PD	2.0000 sec
SCANS	16
ADBIT	16
RGAIN	44
BF	0.01 Hz
T1	0.00
T2	0.00
T3	90.00
T4	100.00
EXMOD	single_pulse.ex2
EXPCM	
IRNUC	1H
IFR	395.88 MHz
IRSET	6.28 KHz
IRFIN	0.87 Hz
IRRPW	115 usec
IRATN	79
DFILE	7memberering without side chain
SF	
LKSET	13.20 KHz
LKFIN	75.7 Hz
LKLEV	0
LGAIN	0
LKPHS	0
LKSIG	0
CSPED	0 Hz
FILDC	
FILDF	
CTEMP	19.2 c
SLVNT	CDCL3
EXREF	7.26 ppm

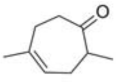

12

Cycloheptenone 12

C:\Documents and Settings\PC-USER\My Documents\Data\TTTTTTTTTTTTTTTTTTTabuchi toshiki\Master thesis\letter\7-memb\TT1-120921-7-memb-C-2.als

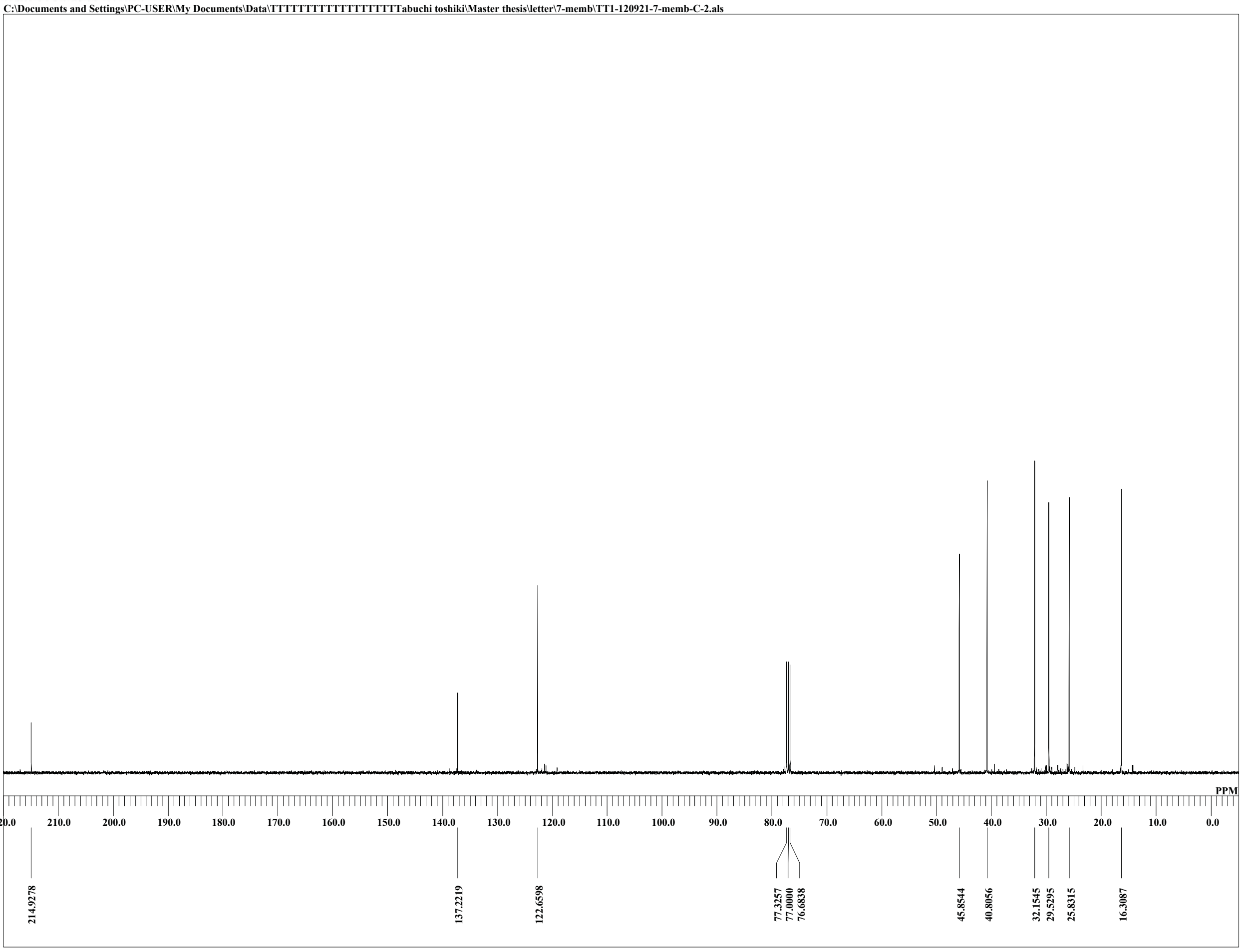

DFILE	TT1-120921-7-memb-C-2.als
COMNT	TT1-120921-7-memb-C
DATIM	21-09-2012 12:44:33
MENUF	
OBNUC	13C
OFR	99.55 MHz
OBFRQ	99.55 MHz
OBSET	5.13 KHz
OBFIN	0.98 Hz
PW1	3.25 usec
DEADT	0.00 usec
PREDL	0.00000 msec
IWT	1.0000 sec
POINT	26214
SPO	26214
TIMES	160
DUMMY	4
FREQU	24999.62 Hz
FLT	125000 Hz
DELAY	20.50 usec
ACQTM	1.0486 sec
PD	2.0000 sec
SCANS	160
ADBIT	16
RGAIN	60
BF	1.00 Hz
T1	0.00
T2	0.00
T3	100.00
T4	100.00
EXMOD	single_pulse_dec
EXPCM	
IRNUC	1H
IFR	395.88 MHz
IRSET	6.28 KHz
IRFIN	0.87 Hz
IRRPW	115 usec
IRATN	79
DFILE	TT1-120921-7-memb-C-2.als
SF	
LKSET	13.20 KHz
LKFIN	75.7 Hz
LKLEV	0
LGAIN	0
LKPHS	0
LKSIG	0
CSPED	0 Hz
FILDC	
FILDF	
CTEMP	24.6 c
SLVNT	CDCL3
EXREF	77.00 ppm

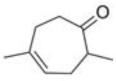

12

TMS-enol ether 13

C:\Documents and Settings\PC-USER\My Documents\Data\TTTTTTTTTTTTTTTTTTTabuchi toshiki\Master thesis\TMS-enolether\TT1-090720-1-173-TMinPhH.als

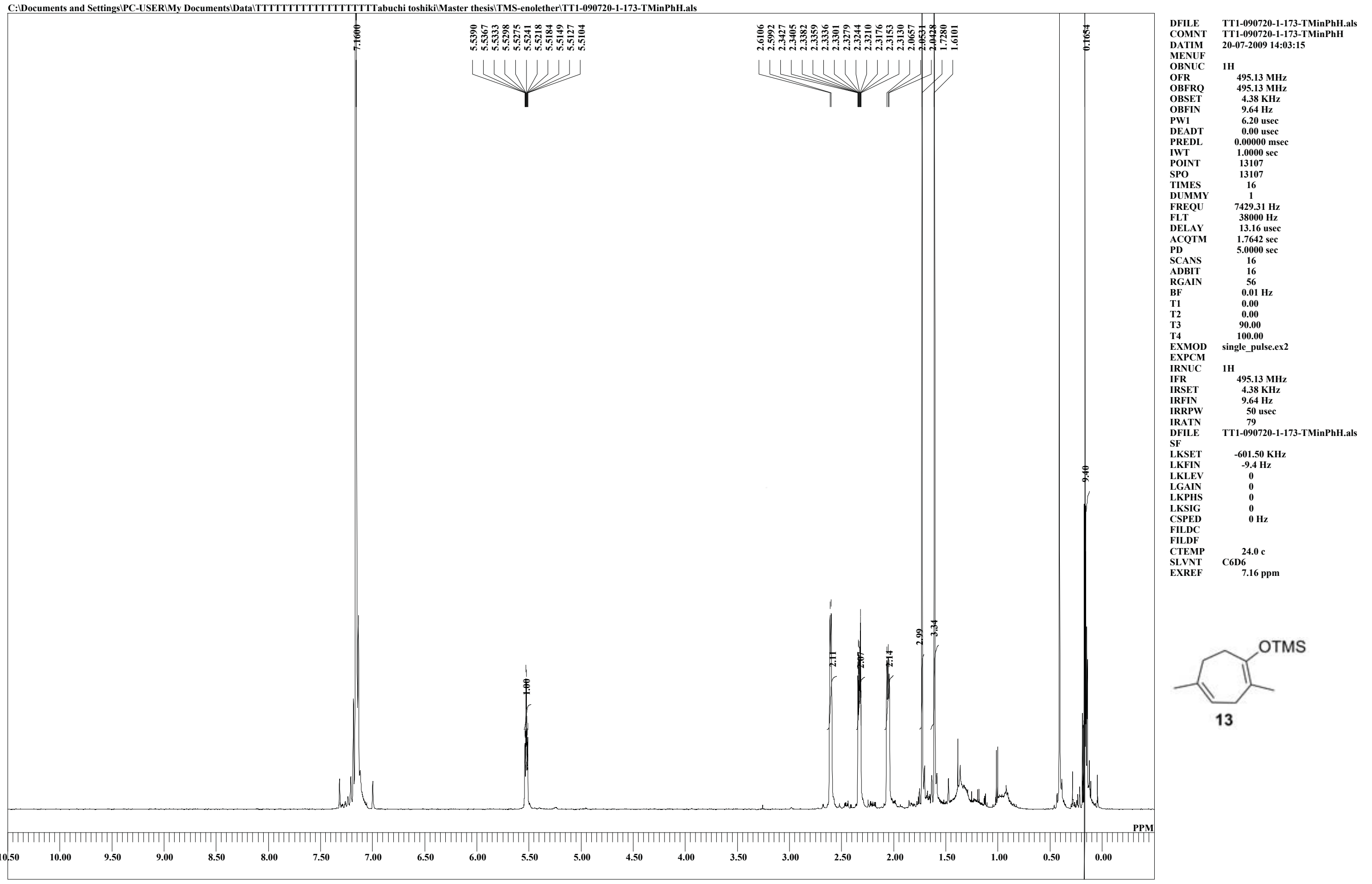

TMS-enol ether 13

G:\PHannou_inoue\Tabuchi Toshiki\ryanodine\DATA\chiral-intermediate\silylenolether\desired\TT1-090720-1-173-TMinPhH-C.als

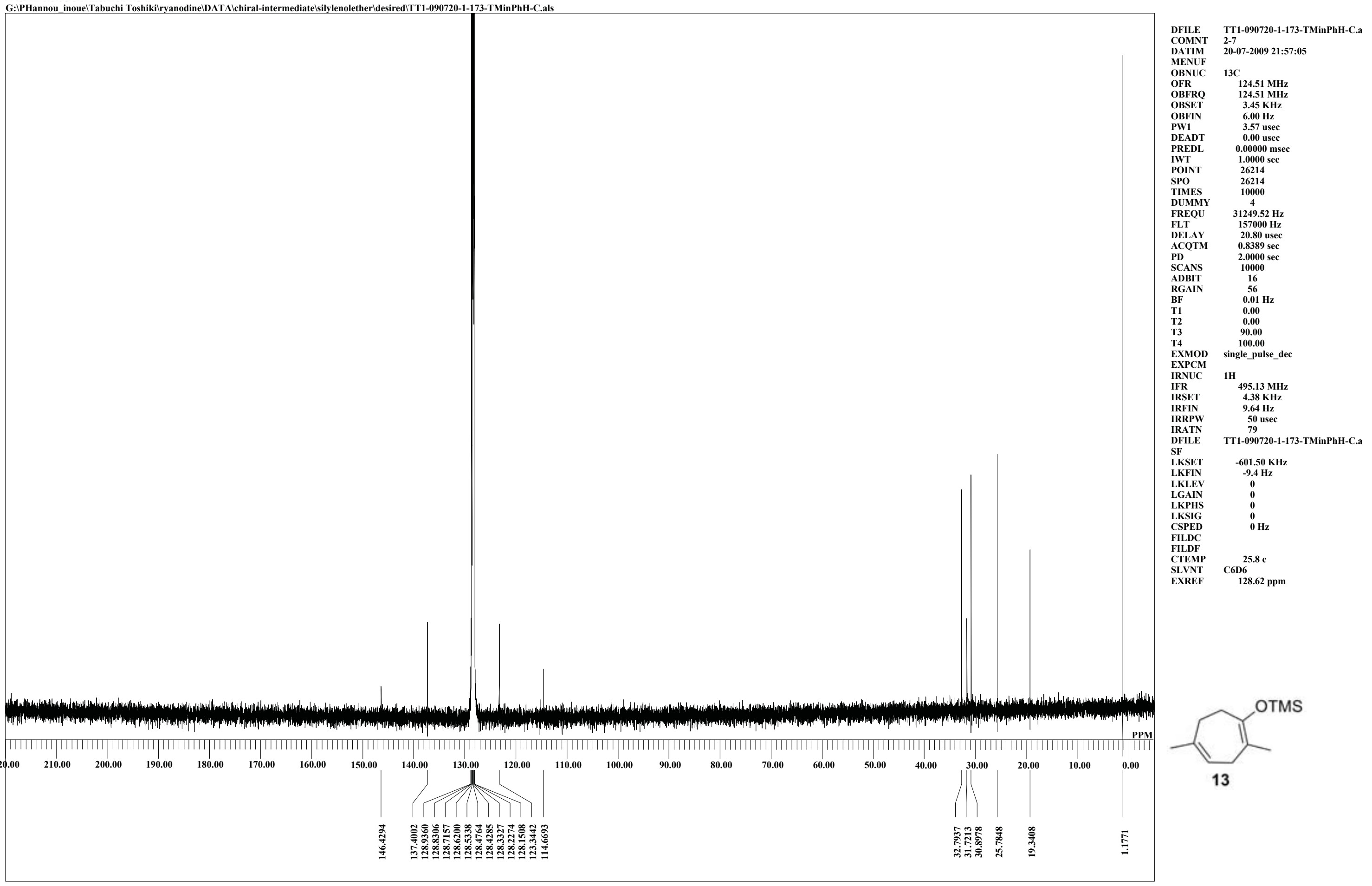

regioisomer of 13

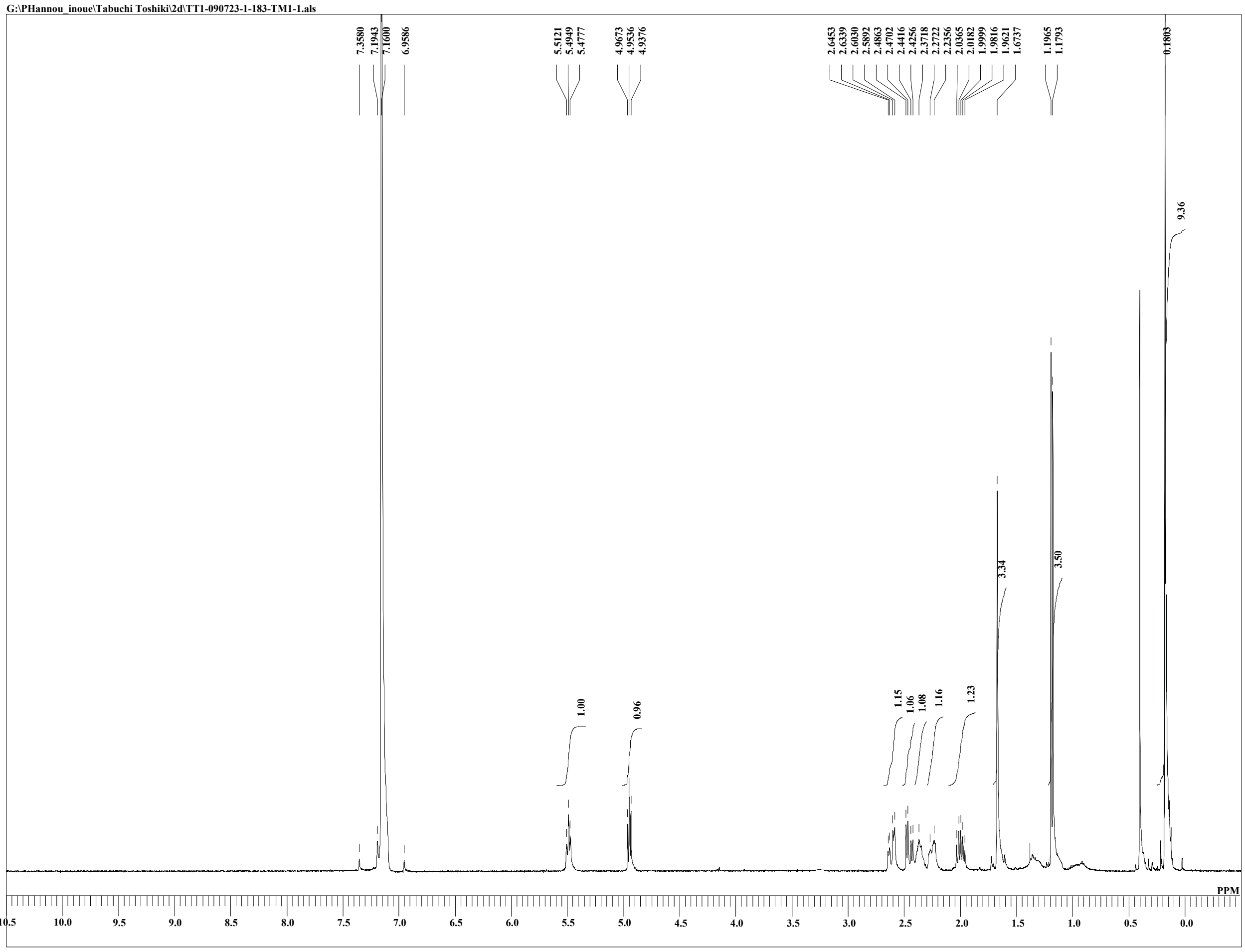

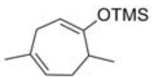

regioisomer of 13

Diene 14

G:\PHannou_inoue\Tabuchi Toshiki\ryanodine\DATA\chiral-intermediate\dienone\dienone(1-195-TM).als

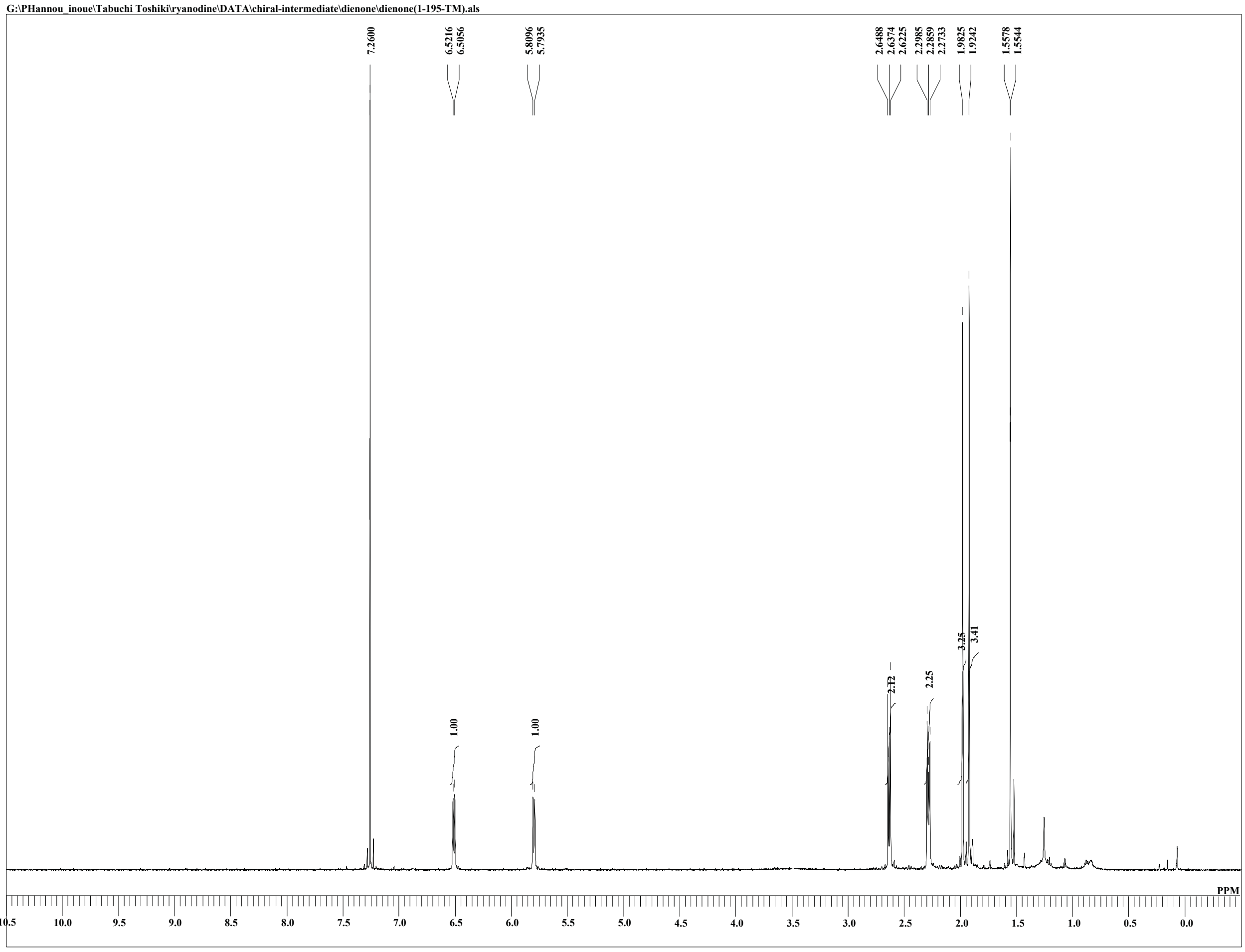

DFILE dienone(1-195-TM).als
COMNT 1-38
DATIM 27-07-2009 22:28:55
MENUF
OBNUC 1H
OFR 495.13 MHz
OBFRQ 495.13 MHz
OBSET 4.38 KHz
OBFIN 9.64 Hz
PW1 6.20 usec
DEADT 0.00 usec
PREDL 0.00000 msec
IWT 1.0000 sec
POINT 13107
SPO 13107
TIMES 16
DUMMY 1
FREQU 7429.31 Hz
FLT 38000 Hz
DELAY 13.16 usec
ACQTM 1.7642 sec
PD 2.0000 sec
SCANS 16
ADBIT 16
RGAIN 60
BF 0.01 Hz
T1 0.00
T2 0.00
T3 90.00
T4 100.00
EXMOD single_pulse.ex2
EXPCM
IRNUC 1H
IFR 495.13 MHz
IRSET 4.38 KHz
IRFIN 9.64 Hz
IRRPW 50 usec
IRATN 79
DFILE dienone(1-195-TM).als
SF
LKSET 748.40 KHz
LKFIN 98.2 Hz
LKLEV 0
LGAIN 0
LKPHS 0
LKSIG 0
CSPED 0 Hz
FILDC
FILDF
CTEMP 24.3 c
SLVNT CDCL3
EXREF 7.26 ppm

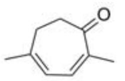

14

Diene 14

G:\PHannou_inoue\Tabuchi Toshiki\ryanodine\DATA\chiral-intermediate\dienone\dienone(2-1-TM-C)-1.als

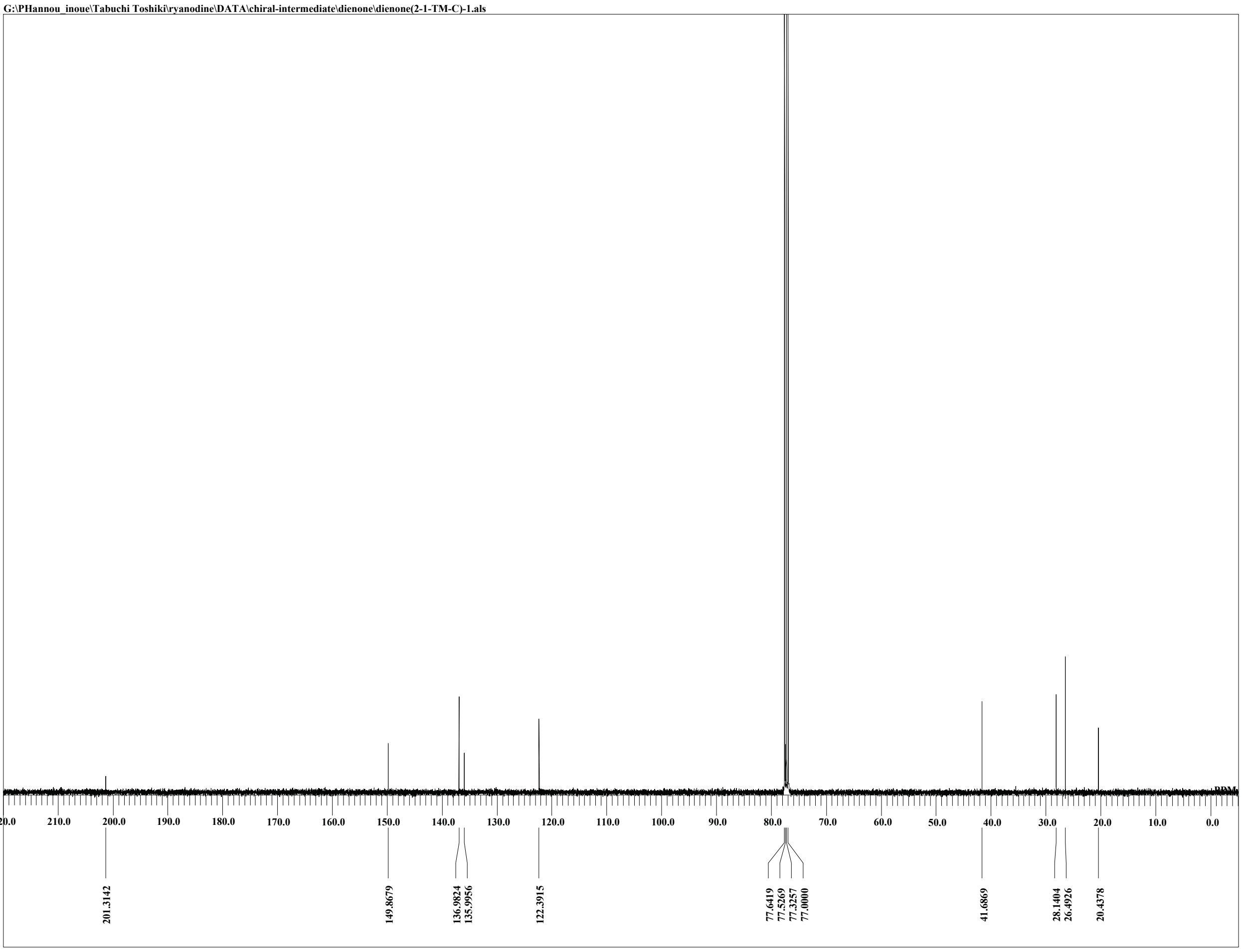

DFILE dienone(2-1-TM-C)-1.als
COMNT 1-38
DATIM 31-07-2009 06:29:11
MENUF
OBNUC 13C
OFR 99.55 MHz
OBFRQ 99.55 MHz
OBSET 5.13 KHz
OBFIN 0.98 Hz
PW1 3.25 usec
DEADT 0.00 usec
PREDL 0.00000 msec
IWT 1.0000 sec
POINT 26214
SPO 26214
TIMES 7000
DUMMY 4
FREQU 24999.62 Hz
FLT 125000 Hz
DELAY 20.50 usec
ACQTM 1.0486 sec
PD 2.0000 sec
SCANS 7000
ADBIT 16
RGAIN 60
BF 0.01 Hz
T1 0.00
T2 0.00
T3 60.00
T4 100.00
EXMOD single_pulse_dec
EXPCM
IRNUC 1H
IFR 395.88 MHz
IRSET 6.28 KHz
IRFIN 0.87 Hz
IRRPW 115 usec
IRATN 79
DFILE dienone(2-1-TM-C)-1.als
SF
LKSET 13.20 KHz
LKFIN 75.7 Hz
LKLEV 0
LGAIN 0
LKPHS 0
LKSIG 0
CSPED 0 Hz
FILDC
FILDF
CTEMP 24.6 c
SLVNT CDCL3
EXREF 77.00 ppm

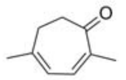

14

Diene 15

G:\PHannou_inoue\Tabuchi Toshiki\ryanodine\DATA\chiral-intermediate\7-memb-alcohol\7member-alcohol-(3-79-TM).als

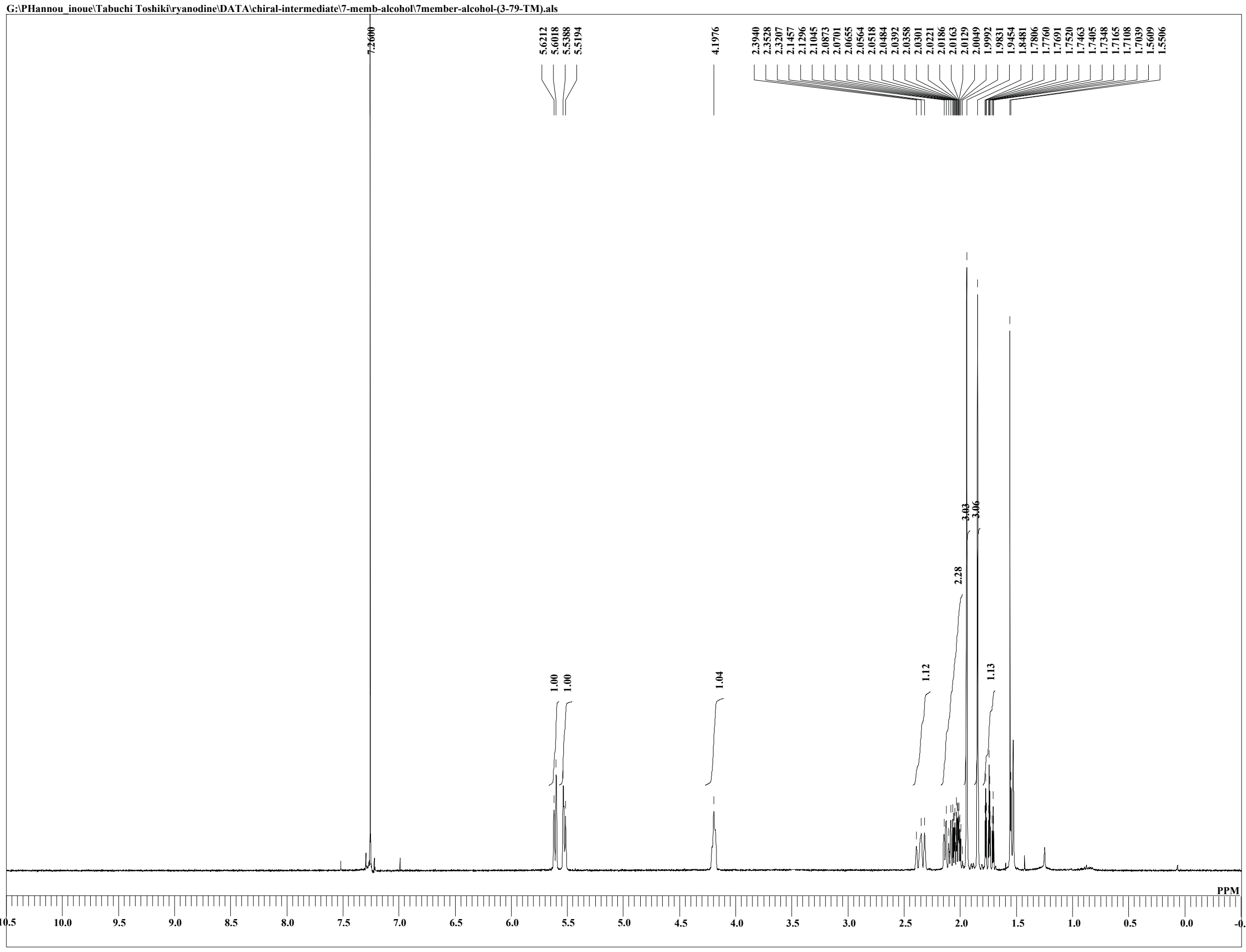

DFILE 7member-alcohol-(3-79-TM).als
COMNT 2-19
DATIM 08-01-2010 10:47:35
MENUF
OBNUC 1H
OFR 395.88 MHz
OBFRQ 395.88 MHz
OBSET 6.28 KHz
OBFIN 0.87 Hz
PW1 6.50 usec
DEADT 0.00 usec
PREDL 0.00000 msec
IWT 1.0000 sec
POINT 13107
SPO 13107
TIMES 16
DUMMY 1
FREQU 5938.15 Hz
FLT 30000 Hz
DELAY 16.68 usec
ACQTM 2.2073 sec
PD 2.0000 sec
SCANS 16
ADBIT 16
RGAIN 50
BF 0.01 Hz
T1 0.00
T2 0.00
T3 90.00
T4 100.00
EXMOD single_pulse.ex2
EXPCM
IRNUC 1H
IFR 395.88 MHz
IRSET 6.28 KHz
IRFIN 0.87 Hz
IRRPW 115 usec
IRATN 79
DFILE 7member-alcohol-(3-79-TM).als
SF
LKSET 13.20 KHz
LKFIN 75.7 Hz
LKLEV 0
LGAIN 0
LKPHS 0
LKSIG 0
CSPED 0 Hz
FILDC
FILDF
CTEMP 18.4 c
SLVNT CDCL3
EXREF 7.26 ppm

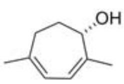

15

Diene 15

G:\PHannou_inoue\Tabuchi Toshiki\ryanodine\DATA\chiral-intermediate\7-memb-alcohol\TT1-100330-diene-OH-C.als

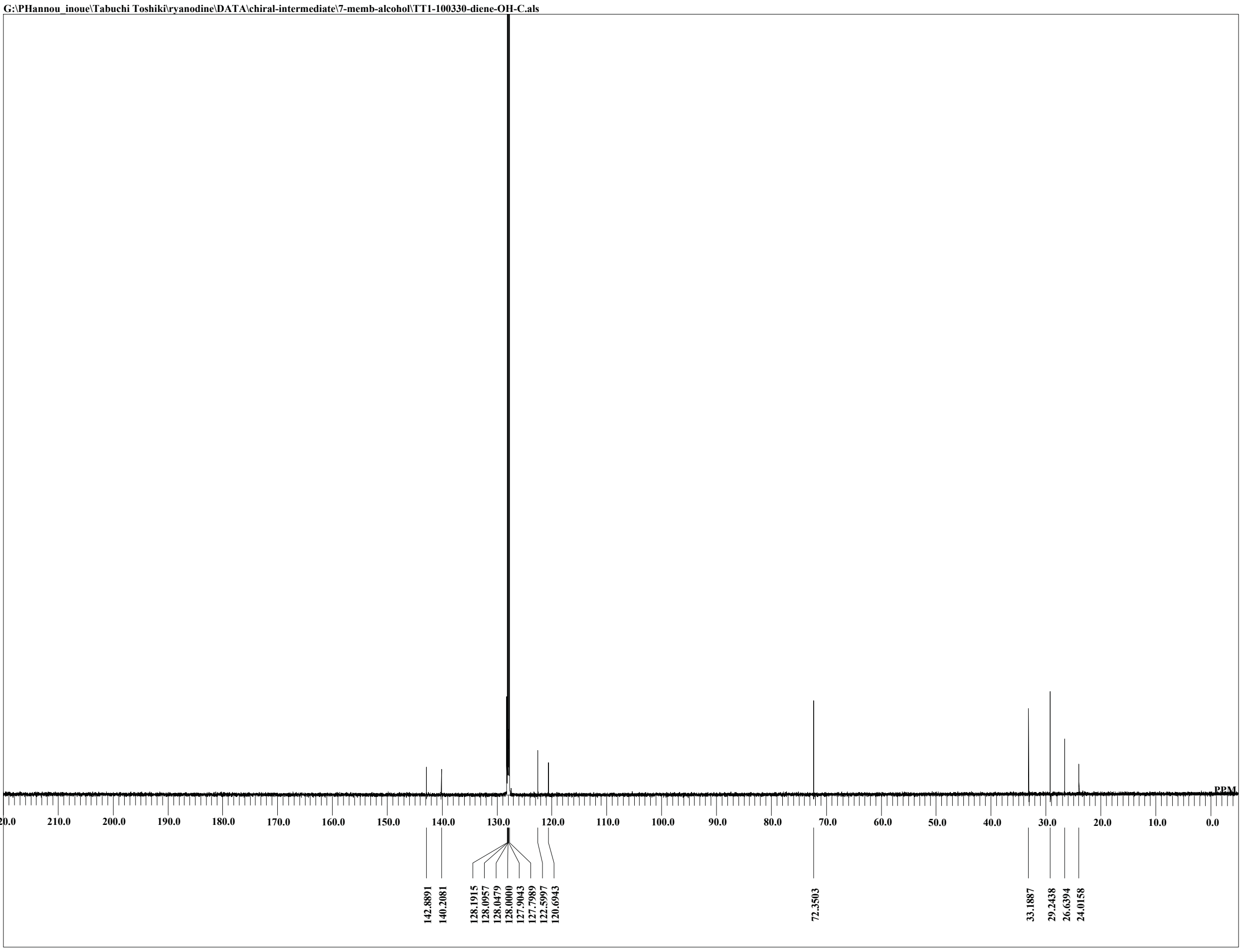

DFILE TT1-100330-diene-OH-C.als
COMNT 2-19
DATIM 30-03-2010 21:09:46
MENUF
OBNUC 13C
OFR 124.51 MHz
OBFRQ 124.51 MHz
OBSET 3.45 KHz
OBFIN 6.00 Hz
PW1 3.25 usec
DEADT 0.00 usec
PREDL 0.00000 msec
IWT 1.0000 sec
POINT 32768
SPO 32768
TIMES 3516
DUMMY 4
FREQU 39062.50 Hz
FLT 157000 Hz
DELAY 20.80 usec
ACQTM 0.8389 sec
PD 2.0000 sec
SCANS 3516
ADBIT 16
RGAIN 58
BF 0.01 Hz
T1 0.00
T2 0.00
T3 90.00
T4 100.00
EXMOD single_pulse_dec
EXPCM
IRNUC 1H
IFR 495.13 MHz
IRSET 4.38 KHz
IRFIN 9.64 Hz
IRRPW 92 usec
IRATN 79
DFILE TT1-100330-diene-OH-C.als
SF
LKSET 748.40 KHz
LKFIN 90.6 Hz
LKLEV 0
LGAIN 0
LKPHS 0
LKSIG 0
CSPED 0 Hz
FILDC
FILDF
CTEMP 19.6 c
SLVNT C6D6
EXREF 128.00 ppm

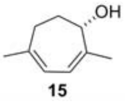

15

Determination of optical purity

C:\Documents and Settings\PC-USER\My Documents\Data\TTTTTTTTTTTTTTTTTTTabuchi toshiki\TT1-110304-6-33-crude-1.jdf

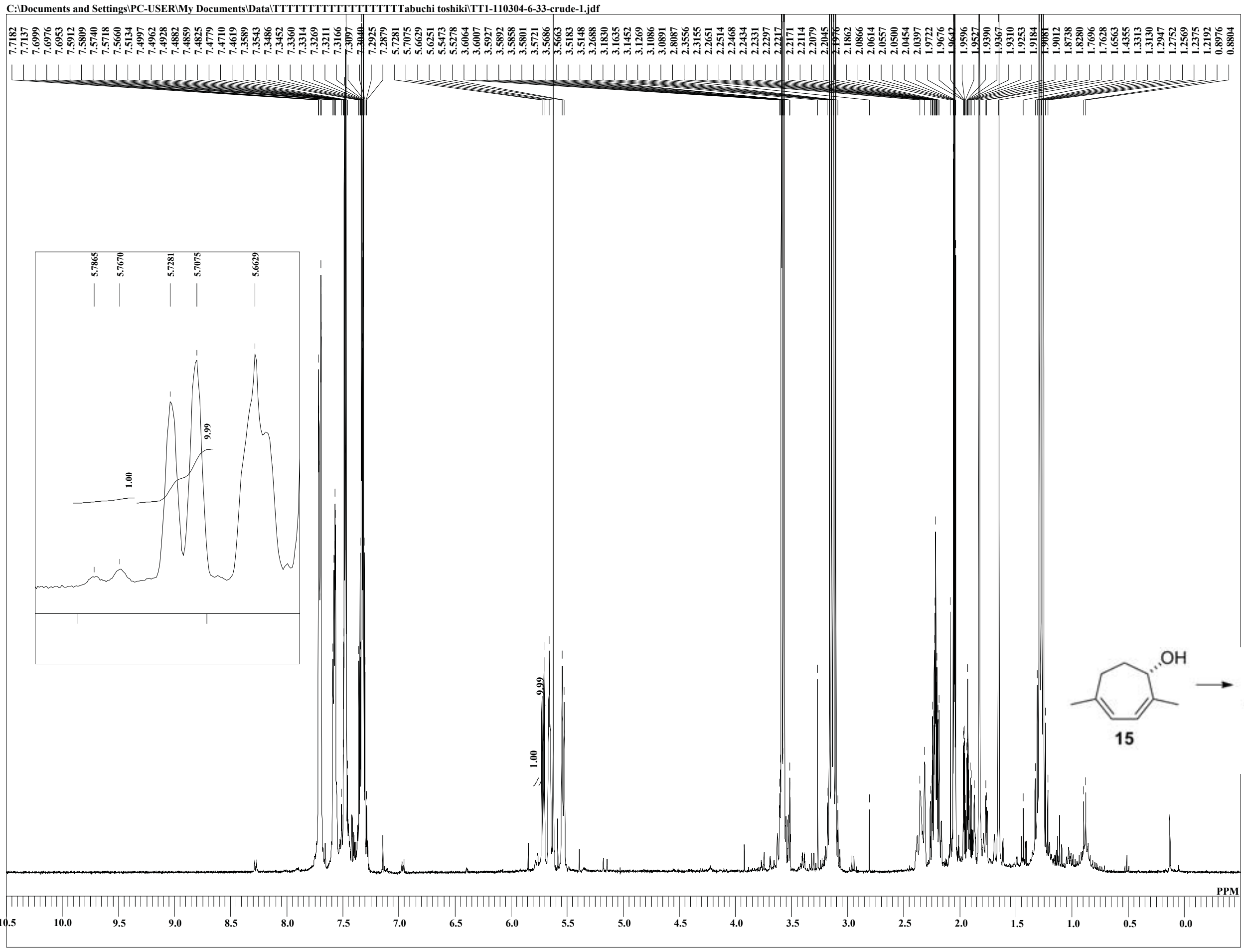

DFILE	TT1-110304-6-33-crude-1.jdf
COMNT	TT1-110304-6-33-crude
DATIM	04-03-2011 10:54:35
MENUF	
OBNUC	1H
OFR	395.88 MHz
OBFRQ	395.88 MHz
OBSET	6.28 KHz
OBFIN	0.87 Hz
PW1	6.50 usec
DEADT	0.00 usec
PREDL	0.00000 msec
IWT	1.0000 sec
POINT	16384
SPO	16384
TIMES	8
DUMMY	1
FREQU	7422.80 Hz
FLT	30000 Hz
DELAY	16.68 usec
ACQTM	2.2073 sec
PD	10.0000 sec
SCANS	8
ADBIT	16
RGAIN	34
BF	0.01 Hz
T1	0.00
T2	0.00
T3	90.00
T4	100.00
EXMOD	single_pulse.ex2
EXPCM	
IRNUC	1H
IFR	395.88 MHz
IRSET	6.28 KHz
IRFIN	0.87 Hz
IRRPW	115 usec
IRATN	79
DFILE	TT1-110304-6-33-crude-1.jdf
SF	
LKSET	12.90 KHz
LKFIN	59.1 Hz
LKLEV	0
LGAIN	0
LKPHS	0
LKSIG	0
CSPED	0 Hz
FILDC	
FILDF	
CTEMP	21.0 c
SLVNT	ACETN
EXREF	2.05 ppm

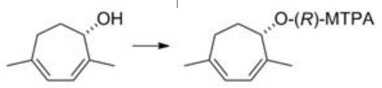

15 → 16b (82% ee)

Diene 7

G:\PHannou_inoue\Tabuchi Toshiki\ryanodine\DATA\chiral-intermediate\diene-OTBS\diene-OTBS(4-45).als

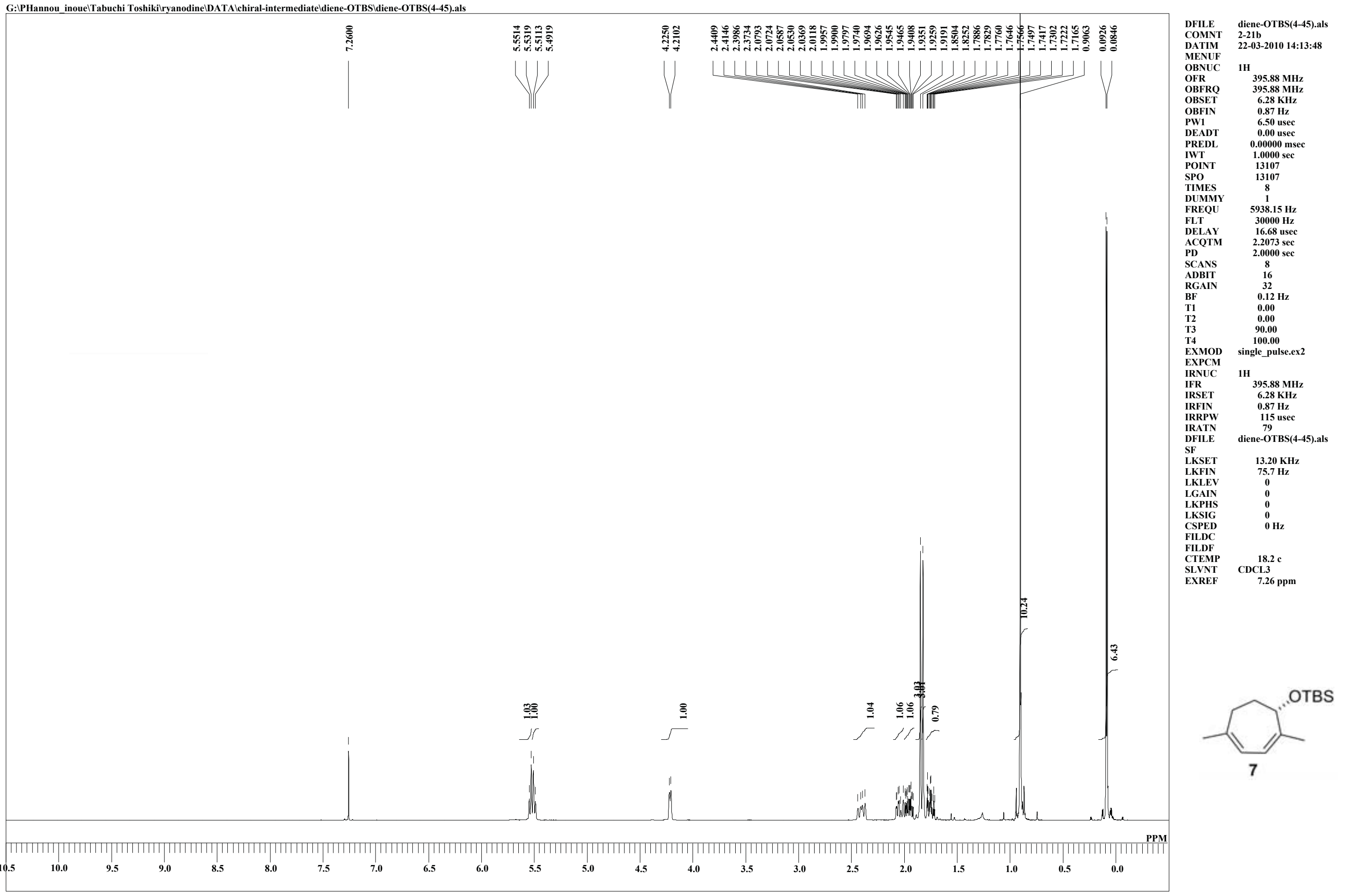

Diene 7

G:\PHannou_inoue\Tabuchi Toshiki\ryanodine\DATA\chiral-intermediate\diene-OTBS\diene-OTBS-C(4-45).als

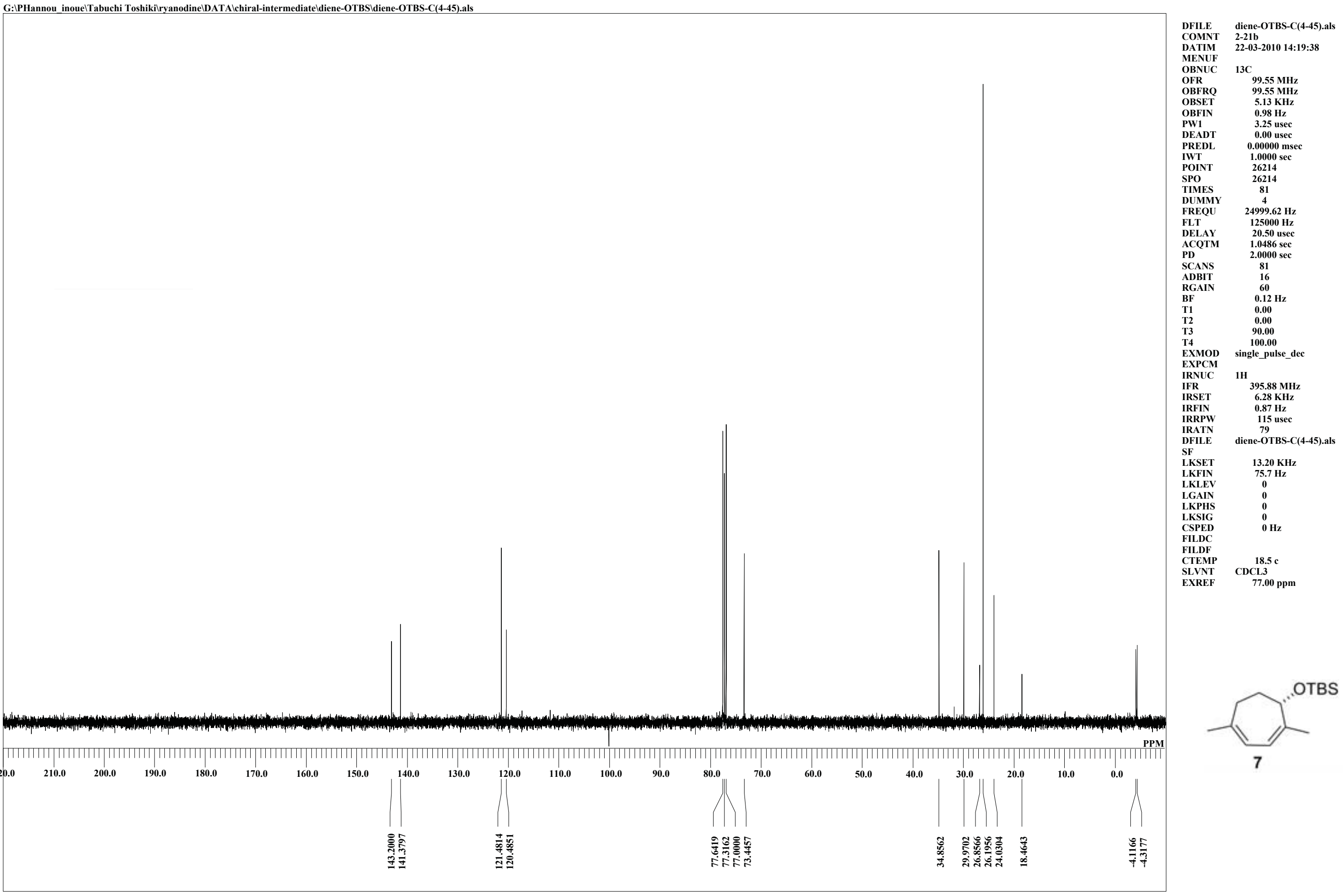

Cyclopropane 17a

C:\Documents and Settings\PC-USER\My Documents\Data\TTTTTTTTTTTTTTTTTTTabuchi toshiki\Master thesis\letter\3-ring\TT1-120515-6-67-3-ring-1.als

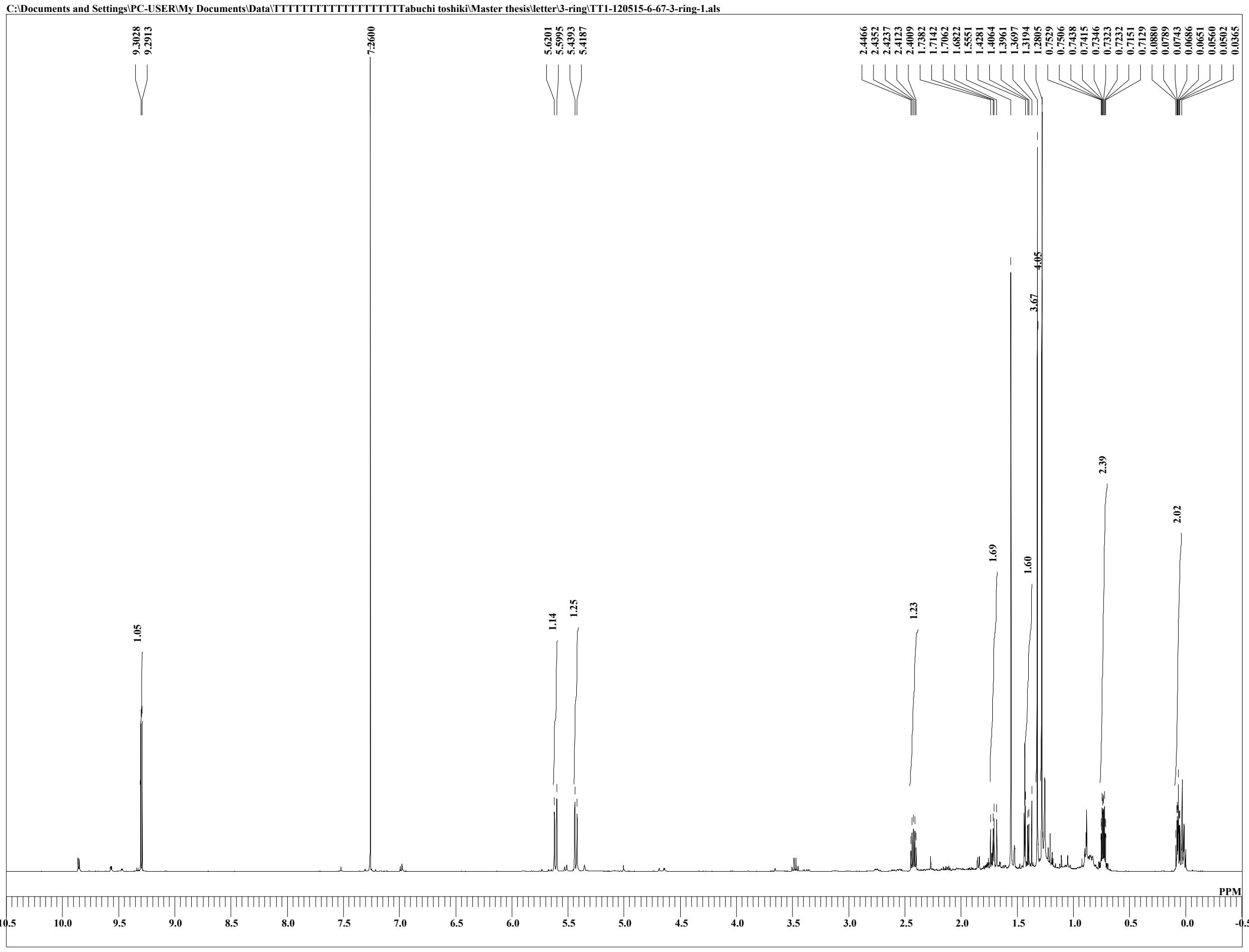

DFILE TT1-120515-6-67-3-ring-1.als
COMNT TT1-120515-6-67-3-ring
DATIM 15-05-2012 23:06:48
MENUF
OBNUC 1H
OFR 395.88 MHz
OBFRQ 395.88 MHz
OBSET 6.28 KHz
OBFIN 0.87 Hz
PW1 6.55 usec
DEADT 0.00 usec
PREDL 0.00000 msec
IWT 1.0000 sec
POINT 13107
SPO 13107
TIMES 32
DUMMY 1
FREQU 5938.15 Hz
FLT 30000 Hz
DELAY 16.68 usec
ACQTM 2.2073 sec
PD 5.0000 sec
SCANS 32
ADBIT 16
RGAIN 38
BF 0.01 Hz
T1 0.00
T2 0.00
T3 90.00
T4 100.00
EXMOD single_pulse.ex2
EXPCM
IRNUC 1H
IFR 395.88 MHz
IRSET 6.28 KHz
IRFIN 0.87 Hz
IRRPW 115 usec
IRATN 79
DFILE TT1-120515-6-67-3-ring-1.als
SF
LKSET 13.20 KHz
LKFIN 75.7 Hz
LKLEV 0
LGAIN 0
LKPHS 0
LKSIG 0
CSPED 0 Hz
FILDC
FILDF
CTEMP 24.3 c
SLVNT CDCL3
EXREF 7.26 ppm

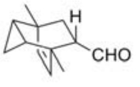

17a

Cyclopropane 17a

C:\Documents and Settings\PC-USER\My Documents\Data\TTTTTTTTTTTTTTTTTTTabuchi toshiki\Master thesis\letter\3-ring\TT1-120515-6-67-3-ring-C-1.als

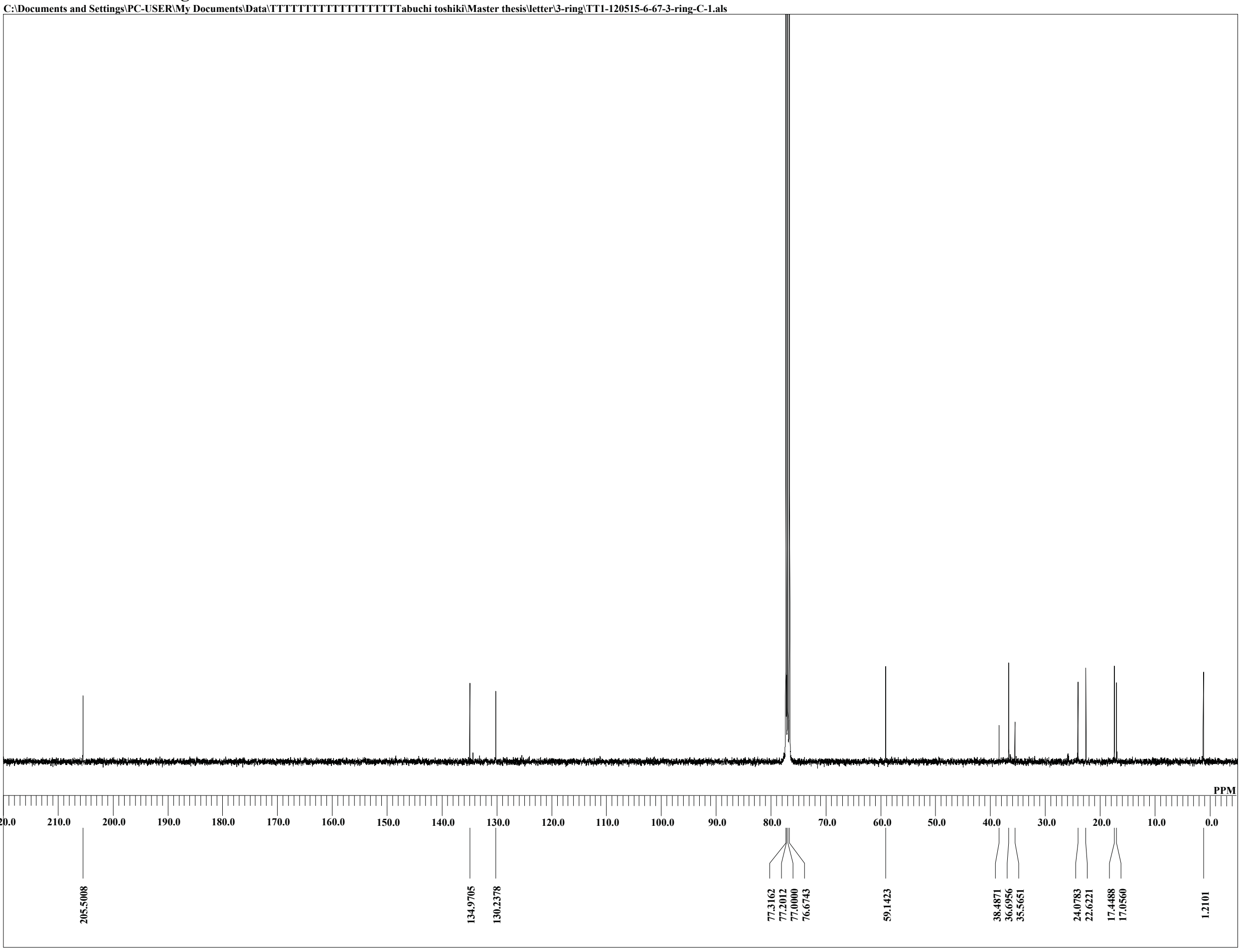

DFILE TT1-120515-6-67-3-ring-C-1.als
COMNT TT1-120515-6-67-3-ring-C
DATIM 16-05-2012 08:26:15
MENUF
OBNUC 13C
OFR 99.55 MHz
OBFRQ 99.55 MHz
OBSET 5.13 KHz
OBFIN 0.98 Hz
PW1 3.67 usec
DEADT 0.00 usec
PREDL 0.00000 msec
IWT 1.0000 sec
POINT 26214
SPO 26214
TIMES 11000
DUMMY 4
FREQU 24999.62 Hz
FLT 125000 Hz
DELAY 20.50 usec
ACQTM 1.0486 sec
PD 2.0000 sec
SCANS 11000
ADBIT 16
RGAIN 60
BF 1.00 Hz
T1 0.00
T2 0.00
T3 90.00
T4 100.00
EXMOD single_pulse_dec
EXPCM
IRNUC 1H
IFR 395.88 MHz
IRSET 6.28 KHz
IRFIN 0.87 Hz
IRRPW 115 usec
IRATN 79
DFILE TT1-120515-6-67-3-ring-C-1.als
SF
LKSET 13.20 KHz
LKFIN 75.7 Hz
LKLEV 0
LGAIN 0
LKPHS 0
LKSIG 0
CSPED 0 Hz
FILDC
FILDF
CTEMP 23.8 c
SLVNT CDCL3
EXREF 77.00 ppm

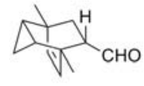

17a

Cycloadduct 8

G:\PHannou_inoue\Tabuchi Toshiki\ryanodine\DATA\chiral-intermediate\-OTBS-DA-acrolein\TT1-100331-4-59-2nd44-1.als

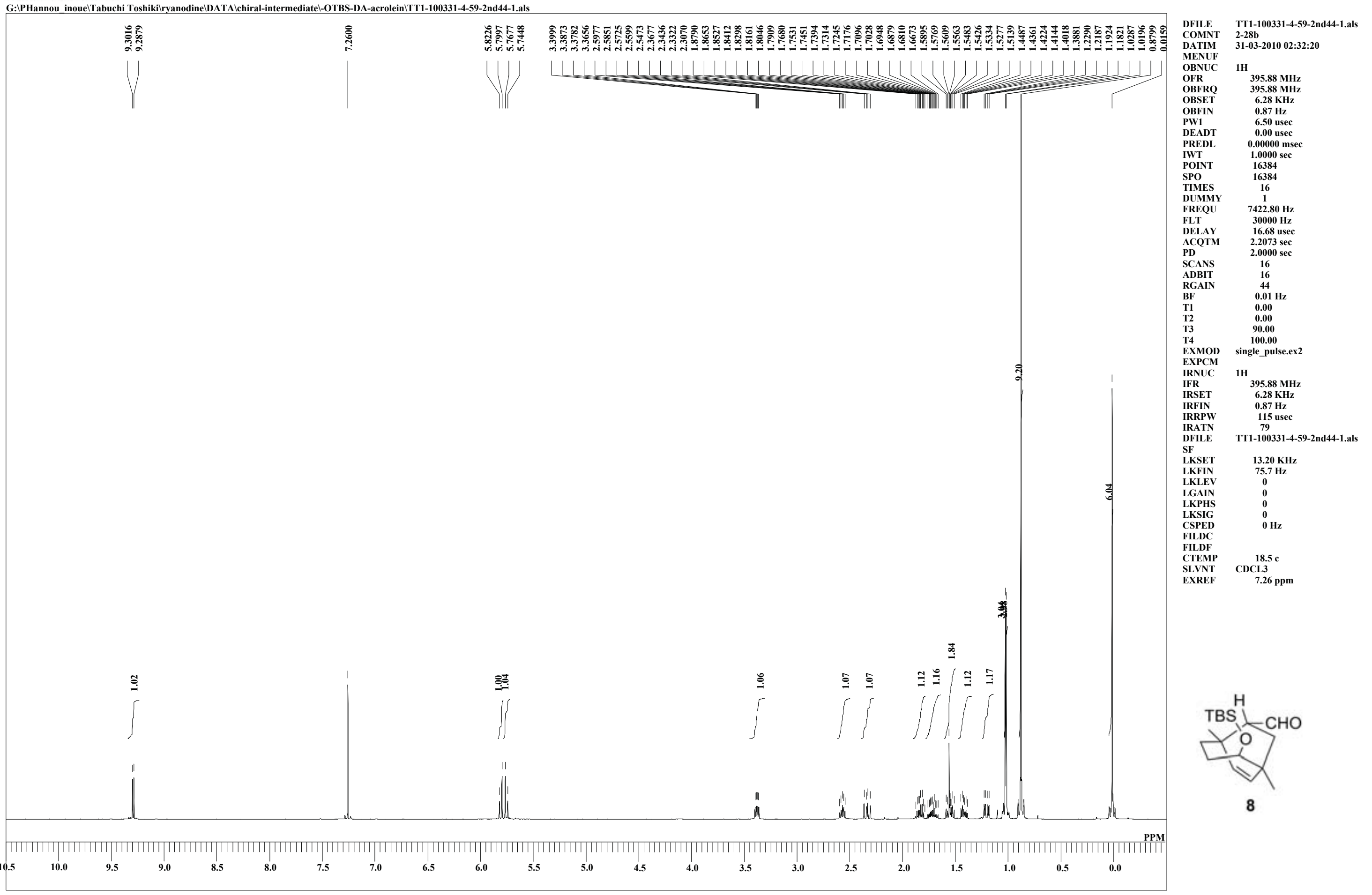

Cycloadduct 8

G:\PHannou_inoue\Tabuchi Toshiki\ryanodine\DATA\chiral-intermediate\-OTBS-DA-acrolein\DA-OTBS-acrolein-C(4-59).als

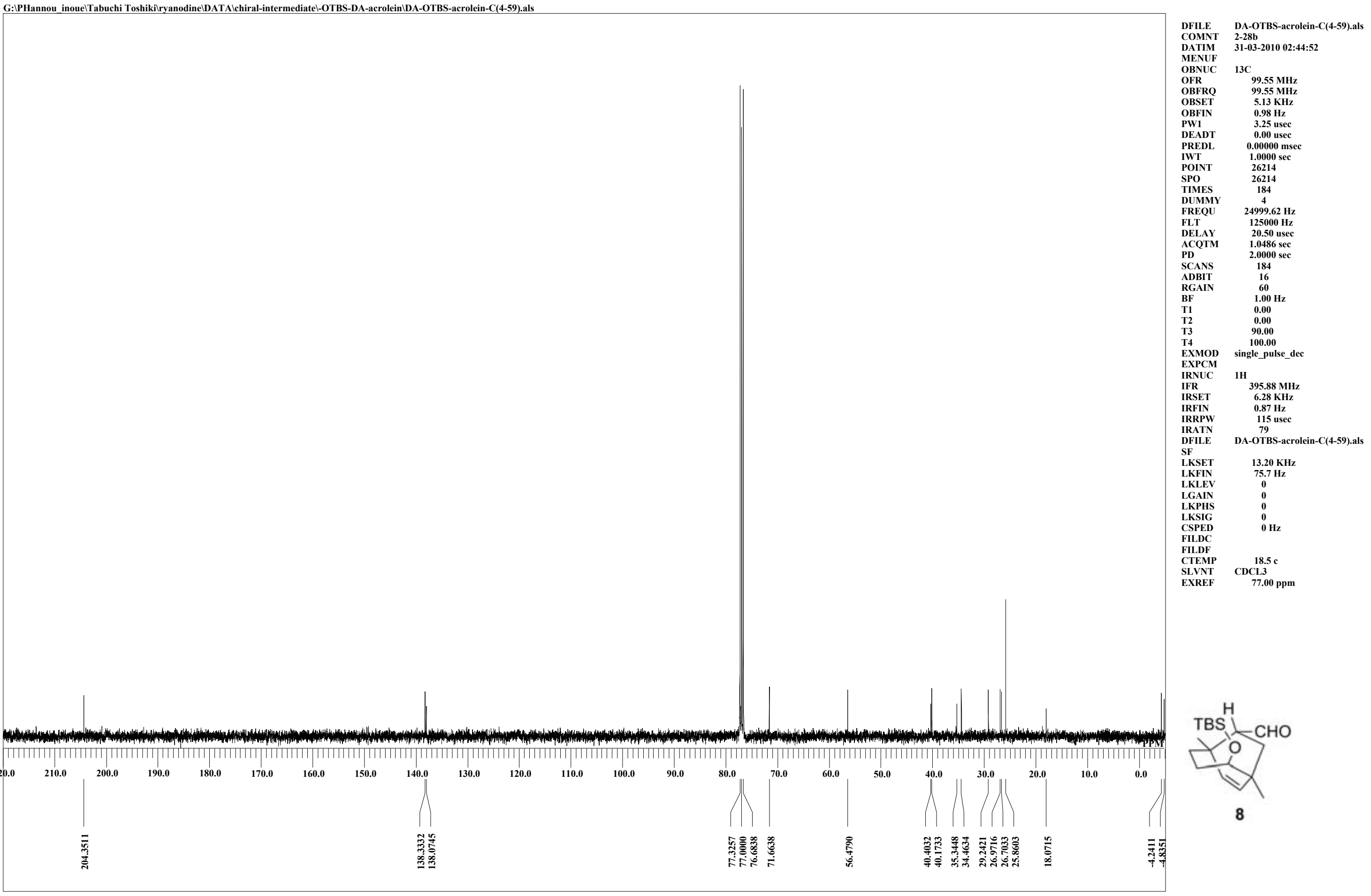

Cycloadduct 20

G:\PHannou_inoue\Tabuchi Toshiki\ryanodine\DATA\chiral-intermediate\-OTBS-DA-acrolein\DA-OTBS-acrolein-radio(4-59).als

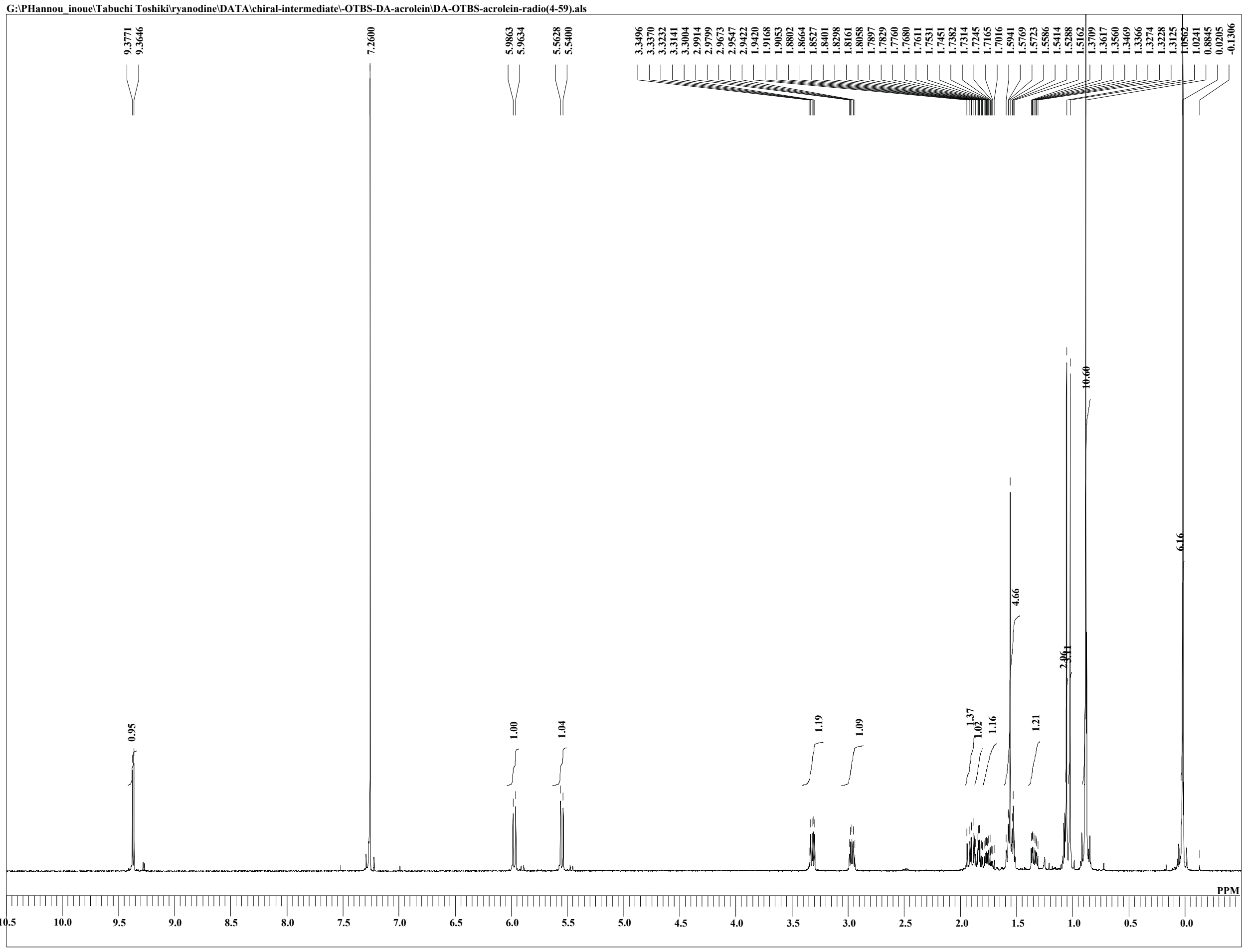

DFILE DA-OTBS-acrolein-radio(4-59).al
COMNT 2-29b
DATIM 31-03-2010 16:57:37
MENUF
OBNUC 1H
OFR 395.88 MHz
OBFRQ 395.88 MHz
OBSET 6.28 KHz
OBFIN 0.87 Hz
PW1 6.50 usec
DEADT 0.00 usec
PREDL 0.00000 msec
IWT 1.0000 sec
POINT 13107
SPO 13107
TIMES 16
DUMMY 1
FREQU 5938.15 Hz
FLT 30000 Hz
DELAY 16.68 usec
ACQTM 2.2073 sec
PD 2.0000 sec
SCANS 16
ADBIT 16
RGAIN 50
BF 0.01 Hz
T1 0.00
T2 0.00
T3 90.00
T4 100.00
EXMOD single_pulse.ex2
EXPCM
IRNUC 1H
IFR 395.88 MHz
IRSET 6.28 KHz
IRFIN 0.87 Hz
IRRPW 115 usec
IRATN 79
DFILE DA-OTBS-acrolein-radio(4-59).al
SF
LKSET 13.20 KHz
LKFIN 75.7 Hz
LKLEV 0
LGAIN 0
LKPHS 0
LKSIG 0
CSPED 0 Hz
FILDC
FILDF
CTEMP 18.1 c
SLVNT CDCL3
EXREF 7.26 ppm

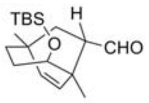

20

Cycloadduct 20

G:\PHannou_inoue\Tabuchi Toshiki\ryanodine\DATA\chiral-intermediate\-OTBS-DA-acrolein\TT1-100331-4-59-radio-C-1.als

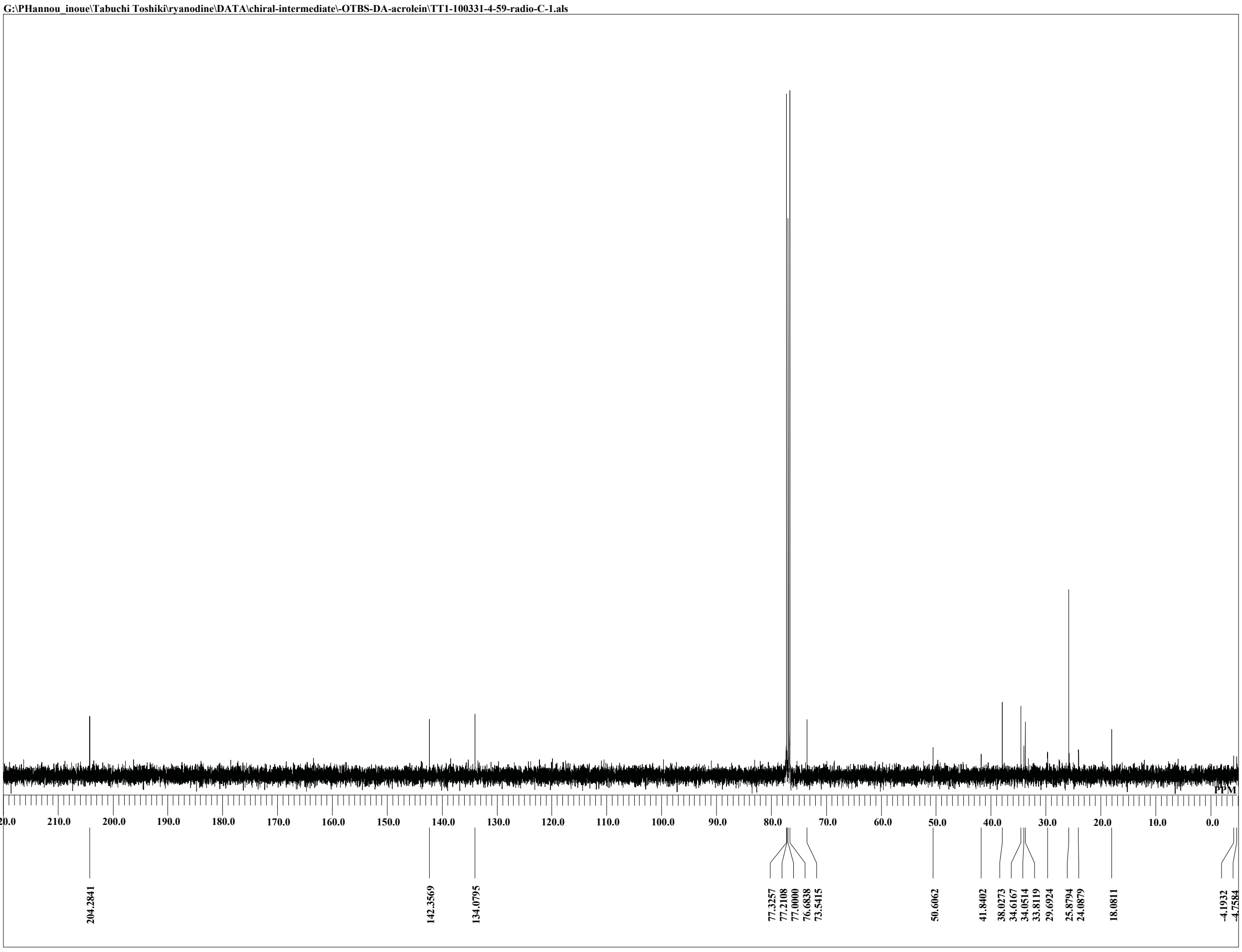

DFILE TT1-100331-4-59-radio-C-1.als
COMNT 2-29
DATIM 31-03-2010 19:26:06
MENUF
OBNUC 13C
OFR 99.55 MHz
OBFRQ 99.55 MHz
OBSET 5.13 KHz
OBFIN 0.98 Hz
PW1 3.25 usec
DEADT 0.00 usec
PREDL 0.00000 msec
IWT 1.0000 sec
POINT 26214
SPO 26214
TIMES 188
DUMMY 4
FREQU 24999.62 Hz
FLT 125000 Hz
DELAY 20.50 usec
ACQTM 1.0486 sec
PD 2.0000 sec
SCANS 188
ADBIT 16
RGAIN 60
BF 0.01 Hz
T1 0.00
T2 0.00
T3 90.00
T4 100.00
EXMOD single_pulse_dec
EXPCM
IRNUC 1H
IFR 395.88 MHz
IRSET 6.28 KHz
IRFIN 0.87 Hz
IRRPW 115 usec
IRATN 79
DFILE TT1-100331-4-59-radio-C-1.als
SF
LKSET 13.20 KHz
LKFIN 75.7 Hz
LKLEV 0
LGAIN 0
LKPHS 0
LKSIG 0
CSPED 0 Hz
FILDC
FILDF
CTEMP 18.4 c
SLVNT CDCL3
EXREF 77.00 ppm

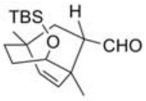

20

Oxime 24a

G:\PHannou_inoue\Tabuchi Toshiki\ryanodine\DATA\chiral-intermediate\7-memb-DA-oxim\TT1-110317-6-45-f14-f12.als

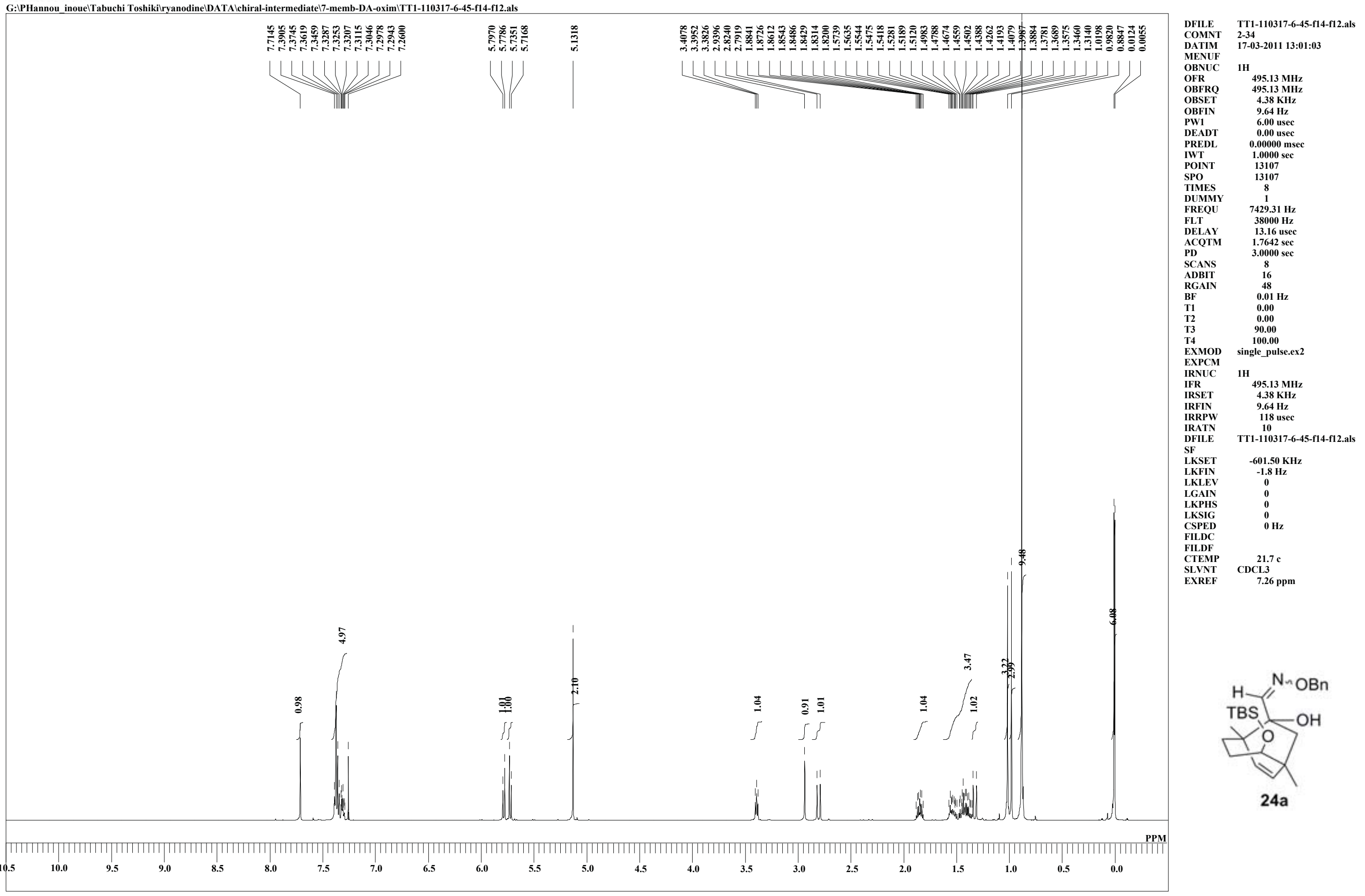

Oxime 24a

G:\PHannou_inoue\Tabuchi Toshiki\ryanodine\DATA\chiral-intermediate\7-memb-DA-oxim\TT1-6-45-f14-f12-C.als

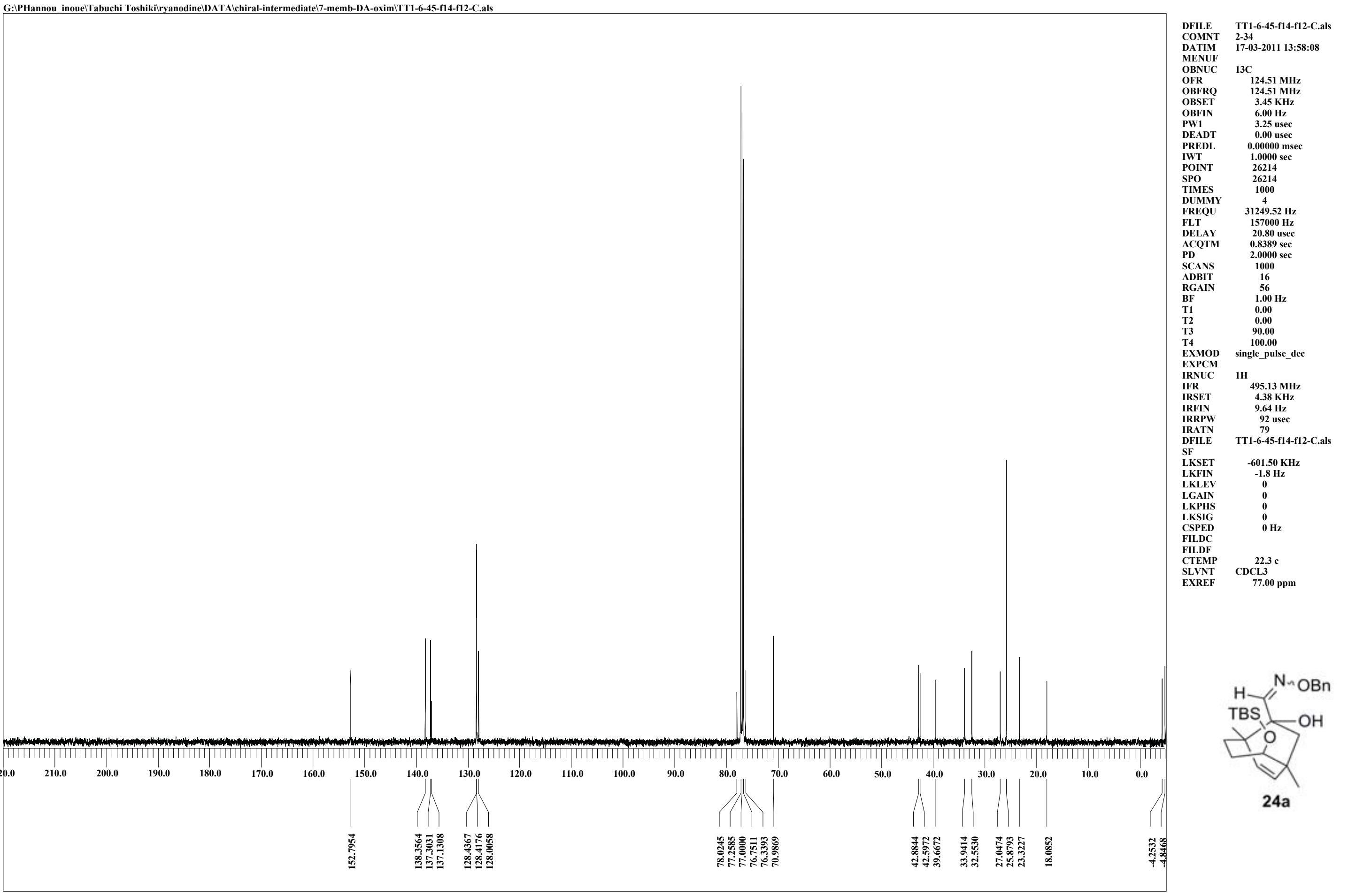

Oxime 24b

C:\Documents and Settings\PC-USER\My Documents\Data\TTTTTTTTTTTTTTTTTTTabuchi toshiki\Master thesis\letter\oxim-dia\24b.als

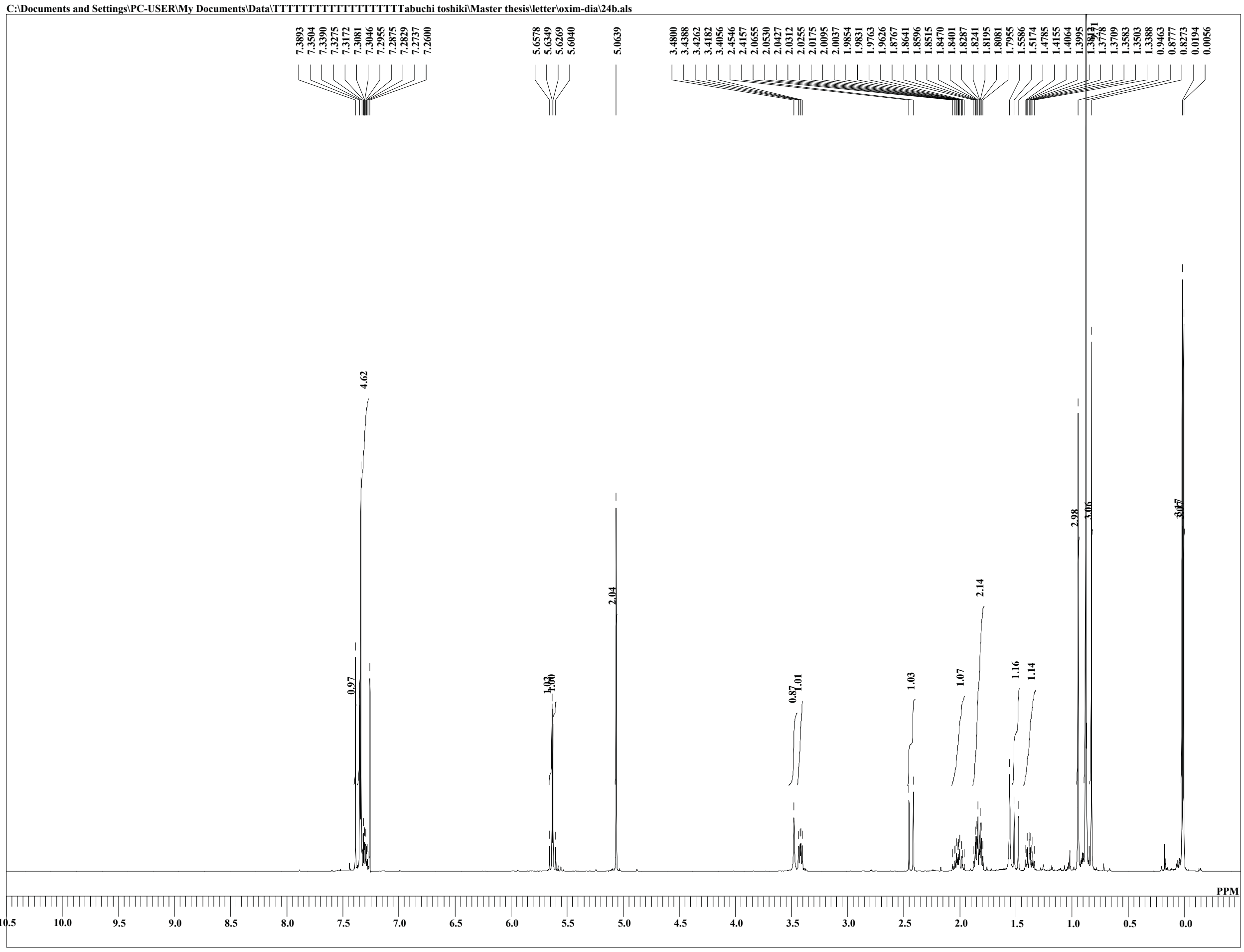

DFILE	24b.als
COMNT	TT1-121010-6-93-tmbB
DATIM	10-10-2012 17:04:25
MENUF	
OBNUC	1H
OFR	395.88 MHz
OBFRQ	395.88 MHz
OBSET	6.28 KHz
OBFIN	0.87 Hz
PW1	6.38 usec
DEADT	0.00 usec
PREDL	0.00000 msec
IWT	1.0000 sec
POINT	13107
SPO	13107
TIMES	8
DUMMY	1
FREQU	5938.15 Hz
FLT	30000 Hz
DELAY	16.68 usec
ACQTM	2.2073 sec
PD	1.5000 sec
SCANS	8
ADBIT	16
RGAIN	40
BF	0.01 Hz
T1	0.00
T2	0.00
T3	90.00
T4	100.00
EXMOD	single_pulse.ex2
EXPCM	
IRNUC	1H
IFR	395.88 MHz
IRSET	6.28 KHz
IRFIN	0.87 Hz
IRRPW	115 usec
IRATN	79
DFILE	24b.als
SF	
LKSET	13.20 KHz
LKFIN	75.7 Hz
LKLEV	0
LGAIN	0
LKPHS	0
LKSIG	0
CSPED	0 Hz
FILDC	
FILDF	
CTEMP	23.6 c
SLVNT	CDCL3
EXREF	7.26 ppm

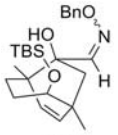

24b

Oxime 24b

C:\Documents and Settings\PC-USER\My Documents\Data\TTTTTTTTTTTTTTTTTTTabuchi toshiki\Master thesis\letter\oxim-dia\TT1-121009-6-93-tmb-C-1.als

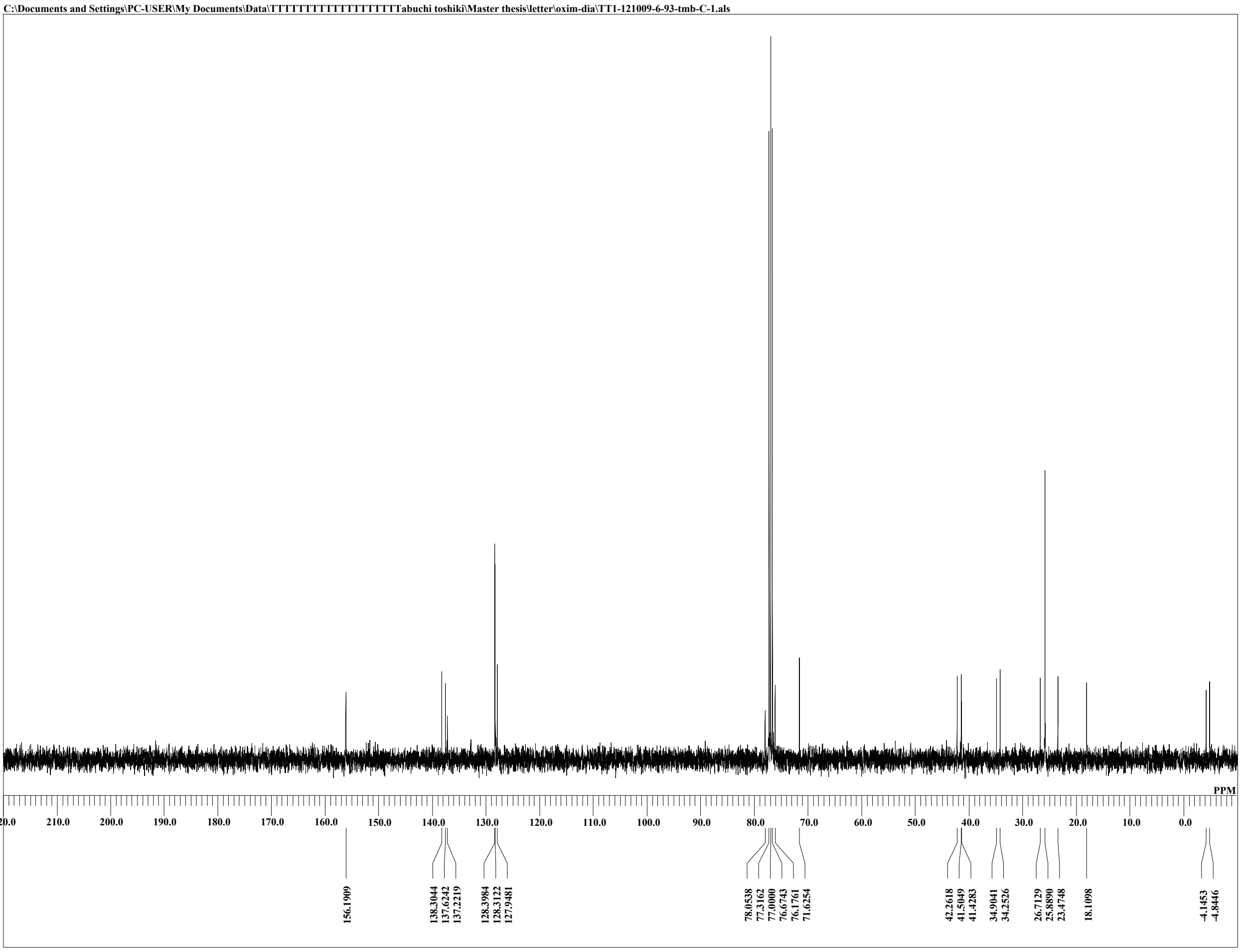

DFILE	TT1-121009-6-93-tmb-C-1.als
COMNT	TT1-121009-6-93-tmb-C
DATIM	09-10-2012 21:08:04
MENUF	
OBNUC	13C
OFR	99.55 MHz
OBFRQ	99.55 MHz
OBSET	5.13 KHz
OBFIN	0.98 Hz
PW1	3.25 usec
DEADT	0.00 usec
PREDL	0.00000 msec
IWT	1.0000 sec
POINT	26214
SPO	26214
TIMES	80
DUMMY	4
FREQU	24999.62 Hz
FLT	125000 Hz
DELAY	20.50 usec
ACQTM	1.0486 sec
PD	2.0000 sec
SCANS	80
ADBIT	16
RGAIN	60
BF	1.00 Hz
T1	0.00
T2	0.00
T3	90.00
T4	100.00
EXMOD	single_pulse_dec
EXPCM	
IRNUC	1H
IFR	395.88 MHz
IRSET	6.28 KHz
IRFIN	0.87 Hz
IRRPW	115 usec
IRATN	79
DFILE	TT1-121009-6-93-tmb-C-1.als
SF	
LKSET	13.20 KHz
LKFIN	75.7 Hz
LKLEV	0
LGAIN	0
LKPHS	0
LKSIG	0
CSPED	0 Hz
FILDC	
FILDF	
CTEMP	24.1 c
SLVNT	CDCL3
EXREF	77.00 ppm

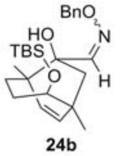

24b

Ketone 27

G:\PHannou_inoue\Tabuchi Toshiki\ryanodine\DATA\chiral-intermediate\ring-expansion\TT1-110323-6-49-TM.als

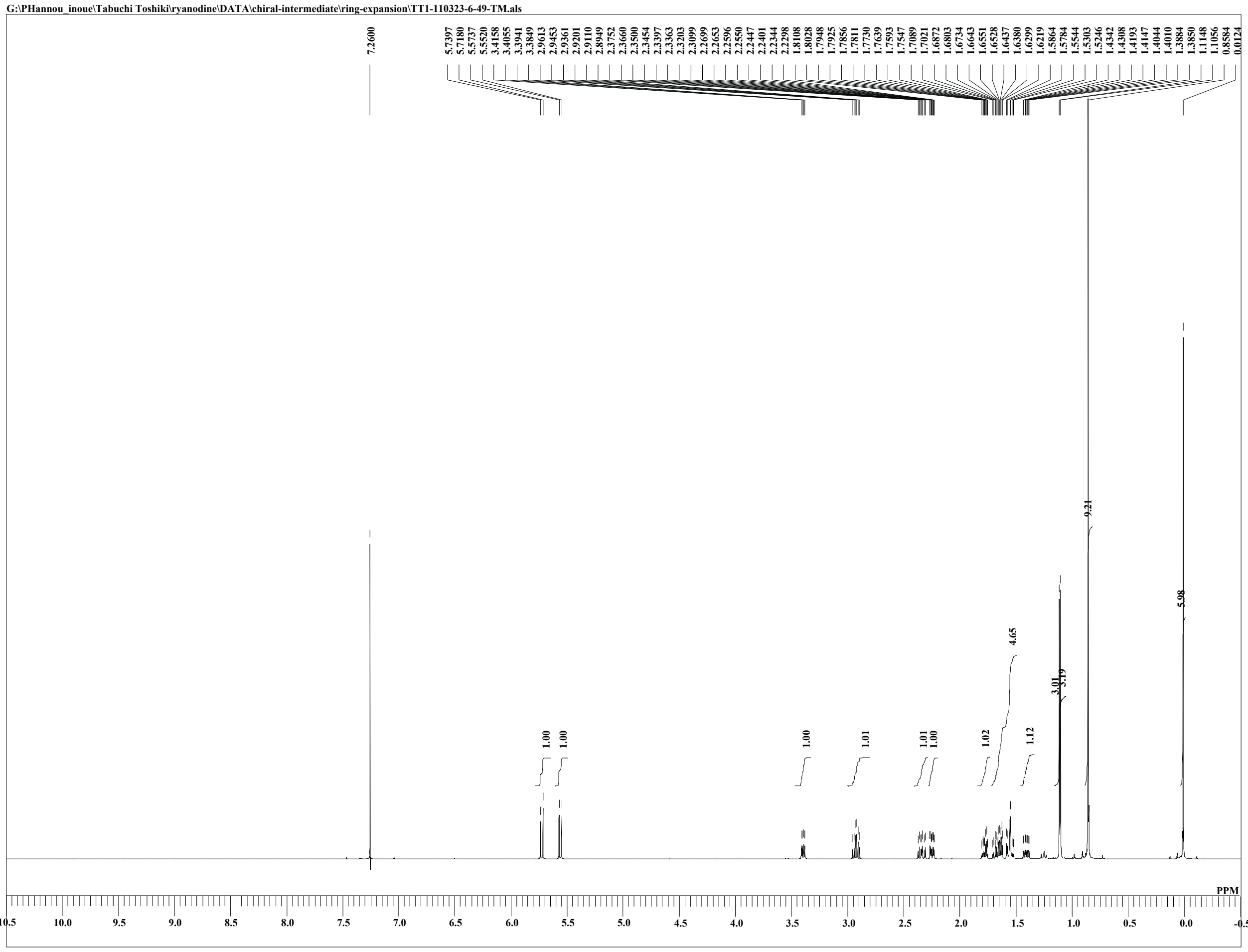

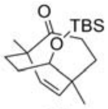

27

Ketone 27

G:\PHannou_inoue\Tabuchi Toshiki\ryanodine\DATA\chiral-intermediate\ring-expansion\TT1-110323-6-49-TM-C.als

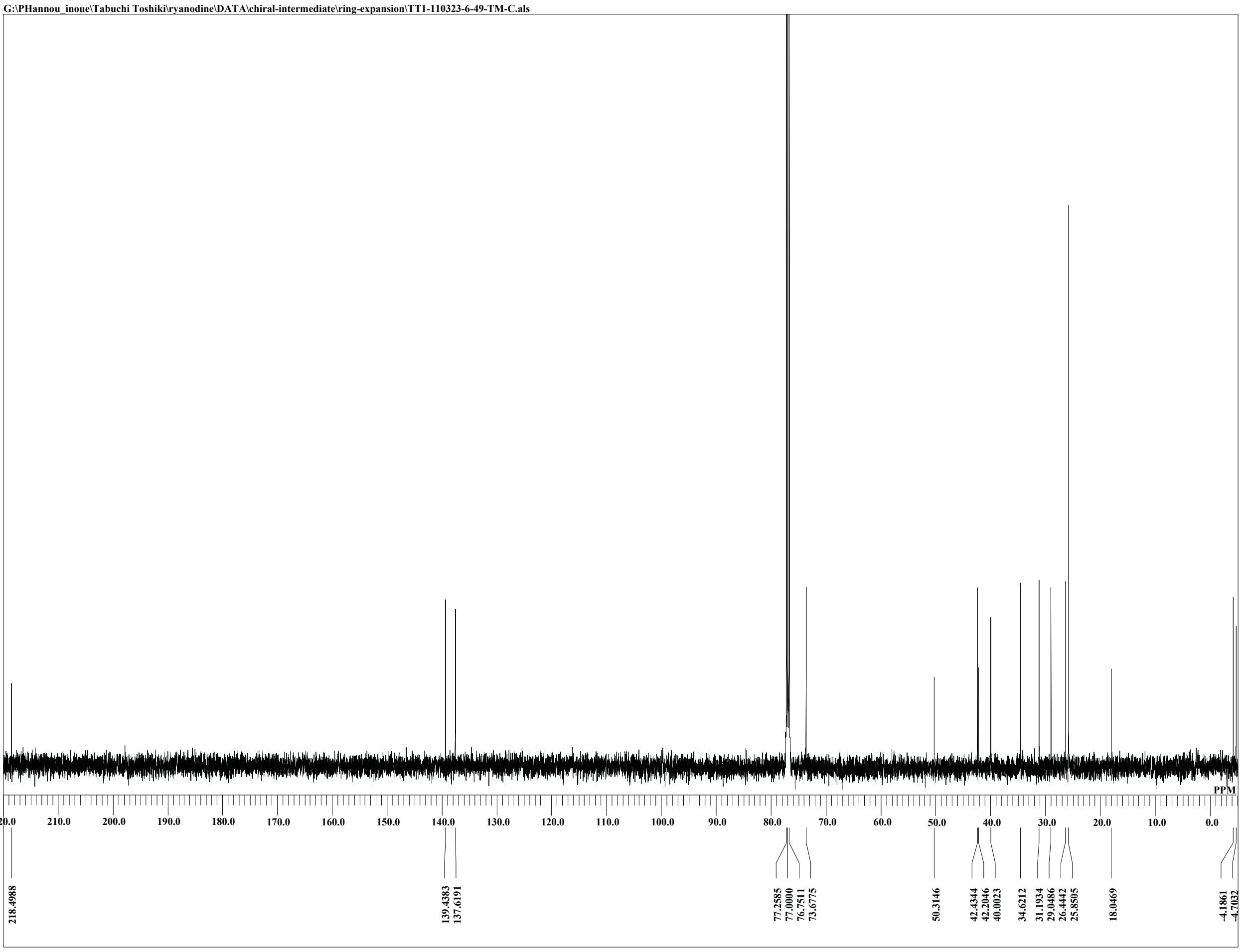

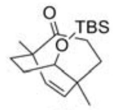

27
